# Plant-associated *Bacillus* mobilizes its secondary metabolites upon perception of the siderophore pyochelin produced by a *Pseudomonas* competitor

**DOI:** 10.1038/s41396-022-01337-1

**Published:** 2022-11-10

**Authors:** Sofija Andrić, Augustin Rigolet, Anthony Argüelles Arias, Sébastien Steels, Grégory Hoff, Guillaume Balleux, Loïc Ongena, Monica Höfte, Thibault Meyer, Marc Ongena

**Affiliations:** 1 Microbial Processes and Interactions Laboratory, Terra Teaching and Research Center, Gembloux Agro-Bio Tech, University of Liège, Gembloux, Belgium; 2 Laboratory of Gene Expression and Cancer, GIGA-MBD, University of Liège, Liège, Belgium; 3 Laboratory of Phytopathology, Department of Plants and Crops, Faculty of Bioscience Engineering, Ghent University, Ghent, Belgium; 4 Present Address: Ecology and Biodiversity, Department of Biology, Utrecht University, Padualaan 8, 3584 CH Utrecht, The Netherlands; 5 Present Address: UMR Ecologie Microbienne, F-69622, University of Lyon, Université Claude Bernard Lyon 1, CNRS, INRAE, VetAgro Sup, Villeurbanne, France

**Keywords:** Microbial ecology, Soil microbiology

## Abstract

*Bacillus velezensis* is considered as model species for plant-associated bacilli providing benefits to its host such as protection against phytopathogens. This is mainly due to the potential to secrete a wide range of secondary metabolites with specific and complementary bioactivities. This metabolite arsenal has been quite well defined genetically and chemically but much remains to be explored regarding how it is expressed under natural conditions and notably how it can be modulated upon interspecies interactions in the competitive rhizosphere niche. Here, we show that *B. velezensis* can mobilize a substantial part of its metabolome upon the perception of *Pseudomonas*, as a soil-dwelling competitor. This metabolite response reflects a multimodal defensive strategy as it includes polyketides and the bacteriocin amylocyclicin, with broad antibiotic activity, as well as surfactin lipopeptides, contributing to biofilm formation and enhanced motility. Furthermore, we identified the secondary *Pseudomonas* siderophore pyochelin as an info-chemical, which triggers this response via a mechanism independent of iron stress. We hypothesize that *B. velezensis* relies on such chelator sensing to accurately identify competitors, illustrating a new facet of siderophore-mediated interactions beyond the concept of competition for iron and siderophore piracy. This phenomenon may thus represent a new component of the microbial conversations driving the behavior of members of the rhizosphere community.

## Introduction

Due to continuous root exudation, the nutrient-enriched rhizosphere compartment of the soil is viewed as one of the richest habitats in terms of microbial abundance and diversity [1]. Bacteria and to a lesser extent fungi are the most dominant forms but other microorganisms such as archaea, oomycetes, nematodes and protists also retain important functions in rhizobiome functioning and for plant health [2–5]. The rhizosphere thus represents a privileged niche for interspecies bacterial interactions, whether they are cooperative or competitive [2, 4, 6].

Thanks to advanced genomics, analytics, and imaging technologies, substantial progress has been made in deciphering the molecular basis of such competitive interactions. It involves signal interference, the release of toxins deployed by contact-dependent delivery systems but also the production of volatiles and small-size soluble metabolites such as quorum sensing molecules, antibiotics at sub-inhibitory concentrations and siderophores lowering iron availability [7–10]. In response to competitor sensing, bacteria adapt their phenotype and stimulate the production of secondary metabolites acting as antimicrobials or impacting developmental traits such as biofilm formation, motility, and sporulation. These boosted metabolites correspond in most cases to known compounds or structural derivatives, but interspecies interactions may also unlock the synthesis of cryptic molecules [11–13].

Several *Bacillus* species belonging to the *B. subtilis* group are ubiquitous habitants of the rhizobiome [2, 14]. It includes *B. velezensis* considered as model for plant-associated bacilli and known to provide benefits to its host in terms of growth promotion and protection against phytopathogens [15–17]. *B. velezensis* differentiates from other species of the *B. subtilis* group by devoting more than 10% of its genome to gene clusters responsible for the synthesis of specialized metabolites [18, 19]. This secondary metabolome is chemically diverse and includes volatile compounds, terpenes, non-ribosomal dipeptides, cyclic lipopeptides (CLPs), and polyketides (PKs), but also ribosomally synthesized and post-translationally modified lantibiotics and bacteriocins [18, 19]. Guided mainly by practical concerns, the research on *Bacillus* bioactive secondary metabolites has so far primarily focused on the characterization of their biological activities in the context of biocontrol of plant diseases, describing their involvement in direct inhibition of phytopathogens and their activity as elicitors of host immunity leading to enhanced systemic resistance toward infection [20]. Nevertheless, from an ecological viewpoint, some of these compounds retain other functions contributing to the persistence of the producing bacteria in natural settings. However, for *B. velezensis*, as for other species, our knowledge about such ecological relevance of secondary metabolites is still limited and many developmental and signaling functions in multitrophic interactions obviously remain to be discovered [13, 21].

As for other bacterial species, it is assumed that microbial interaction is a major biotic factor modulating the production of bioactive secondary metabolites by *Bacillus*. This has been illustrated in several studies reporting an enhanced production of the antifungal CLPs iturin and fengycin by *B. velezensis* upon perception of a range of soil-dwelling fungi and oomycetes as reviewed previously [22]. Bacilli also associate with plant beneficial arbuscular mycorrhizal fungi but little is known about molecular signaling and there is no indication about possible modulation of secondary metabolites [23]. Some recent reports have described how soil bacilli modulate their behavior when facing bacterial competitors, but almost exclusively describing the effect on developmental traits such as social motility, biofilm formation, and sporulation (reviewed in [22]) or adaptative strategies [24, 25]. However, so far, there are only few papers reporting possible modulation of secondary metabolite production by *Bacillus* upon interbacterial interactions. Some enhanced expression of genes responsible for the synthesis of surfactin, bacilysin, or iturin has been observed upon co-cultivation with bacterial phytopathogens. However, no correlation between such transcriptional stimulation and higher production of the corresponding metabolites has been reported in these works nor in the context of interaction between *Bacillus* and other plant beneficials [26, 27].

In this work, we primarily wanted to evaluate the potential of *B. velezensis* in modulating its secondary metabolome upon perception of a bacterial competitor isolated from a similar rhizosphere niche. We studied pairwise interaction in a simplified bipartite system adequate for uncovering cell-cell communication cues. We performed most experiments in contact-independent settings, in order to investigate cross-talks at distance, i.e. mediated by the perception of diffusible compounds [6, 7, 13, 28]. The *Pseudomonas sessilinigenes* species used here belongs to the *P. fluorescens* group, *P. protegens* subgroup, and was selected as a plant beneficial but highly competitive challenger commonly encountered in rhizobiomes [29]. It also retains the potential to synthesize a wide array of bioactive secondary metabolites, allowing to investigate the chemical interplay between both species [29–32]. By combining molecular and analytical methods with mutational approaches, we observed that *B. velezensis* mobilizes a substantial part of its secondary metabolome by chemically sensing the *Pseudomonas* competitor. As it includes several polyketides and the bacteriocin amylocyclicin, such metabolite response correlates with an enhanced global antibacterial potential. Production of surfactin is also stimulated upon interaction which may contribute to rhizosphere fitness since it favors biofilm formation and motility [33]. We also wanted to decipher the mechanism leading to induction and identified the *Pseudomonas* secondary siderophore (enantio-)pyochelin (E-PCH) as a signal specifically perceived by *Bacillus*. This points out a new role for the siderophore in interspecies chemical conversation signaling. The potential benefits for *Bacillus* of such chelator-sensing phenomenon in microbial interactions is also discussed.

## Material and methods

### Bacterial strains and growth conditions

Strains and plasmids used in this study are listed in Table S1. All bacteria were cultured overnight in Lysogeny Broth (LB) (10 g L^−1^ NaCl, 5 g L^−1^ yeast extract and 10 g L^−1^ tryptone) medium, at 30 °C. After being washed three times in peptone water, bacteria were used for the experimental setup. *B. velezensis* strains were grown at 30 °C in half diluted root exudate-mimicking medium as described previously [34]. *Pseudomonas* strains were cultured in recomposed exudate medium and casamino acid liquid medium (10 g L^−1^ casamino acid, 0.3 g L^−1^ K_2_HPO_4_, 0.5 g L^−1^ MgSO_4_ and pH = 7), at 30 °C. The phytopathogenic bacterial strains were grown in LB liquid medium, at 30 °C.

### Construction of deletion mutants

Plasmids and primers used in this study are presented in Table S1 and S2 respectively. For *B. velezensis* GA1, All deletion mutants were created by marker replacement, following a protocol previously described [35]. Briefly, 1 kb of the upstream region of the targeted gene, antibiotic marker (chloramphenicol or phleomycin cassette) and downstream region of the targeted gene were PCR amplified with appropriate primers (Table S2). To obtain the DNA fragment containing three aforementioned components, the overlap PCR has been used. The modified cells were further selected according to chloramphenicol resistance (phleomycin resistance for double mutants) on LB medium. All gene deletions were verified by PCR with the specific UpF and DwR primers and by the loss of production of the corresponding bioactive secondary metabolites.

Pyoverdine and E-PCH mutants of *P. sessilinigenes* CMR12a were created by the I-SceI endonuclease system and the pEMG, vector as described previously [35, 36]. Briefly, the upstream and downstream regions flanking the *pvdI* (*C4K39_6027*) or the *pchA* (*C4K39_5481*) genes were amplified by PCR, connected via overlap PCR and introduced into the pEMG vector, while using kanamycin as an antibiotic marker. The resulting plasmid was integrated into the CMR12a chromosome by conjugation, via homologous recombination. Further, the kanamycin (25 µg/mL) resistant cells were selected on solid, King B plates and transformed by electroporation with the pSW-2 plasmid (harboring the I-SceI system and gentamycin). Gentamycin (20 µg/mL) resistant colonies on the plates were transmitted to King’s B medium with and without kanamycin to verify the loss of kanamycin resistance. CMR12a mutants were confirmed by PCR with the appropriate UpF and DwR primers (Table S2) and via the loss of E-PCH and pyoverdine production.

### Dual interactions

*B. velezensis* cells were diluted in 2 mL of liquid root exudate-mimicking medium to a final OD_600nm_ of 0.1, in which 2% (v/v) of CMR12a cell-free supernatant was added while the control was supplemented with 2% (v/v) of distilled water. The detailed procedure of CMR12a cell-free supernatant preparation is described in Supplementary Methods. *B. velezensis* liquid cultures were then orbitally shaken in an incubator at 300 rpm, at 30 °C for 24 h (if not indicated differentially). Afterwards, 2 mL of the *Bacillus* culture supernatants were sampled, centrifuged at 5000 rpm at room temperature (approx. 22 °C) for 10 min to extract supernatants and collect the cells. Further, the supernatants were filter-sterilized (0.22 µm) and used for analytical analysis of bioactive secondary metabolites and antibacterial assays. The remaining cells after supernatant collection were stored at −80 °C to avoid RNA degradation, until performing RT-qPCR analysis. The RT-qPCR analysis procedure is described in detail in Supplementary Methods.

### Antimicrobial activity assays

Antibacterial activity of the GA1 wild-type or GA1 mutants’ supernatants was tested against *Xanthomonas campestris* and *Clavibacter michiganensis*. The activity of GA1 supernatants was quantified in microtiter plates (96-well) filled with 250 µL of LB liquid medium, inoculated at OD_600nm_ = 0.1 with *X. campestris* or *C. michiganensis* and supplemented with 2% or 6% (v/v) of the supernatants, respectively. The activity of GA1 supernatants was estimated by measuring the pathogen OD_600nm_ every 30 min for 24 h with a Spectramax (Molecular Devices, Wokingham, UK), continuously shaken at 150 rpm, at 30 °C. Two independent assays each, involving three technical repetitions were performed. For determining the activity of GA1 supernatants on a solid medium, 5 µL of the supernatant was applied to a sterile paper disk (5 mm diameter). After drying, disks were placed on solid LB square plates previously inoculated with a confluent layer of *X. campestris*, *C. michiganensis, Pectobacterium carotovorum, Pseudomonas fuscovaginae*, *Pseudomonas cichorii, Agrobacterium tumefaciens*, or *Rhodococcus fascians*. As a negative control, the same volume of LB liquid medium was used. Plates were incubated at 25 °C for 48 h. Three repetitions were done, and the inhibition zones was measured from the edge of the paper discs to the edge of the inhibition zone.

### Secondary metabolite analysis

**Fig. 1:**
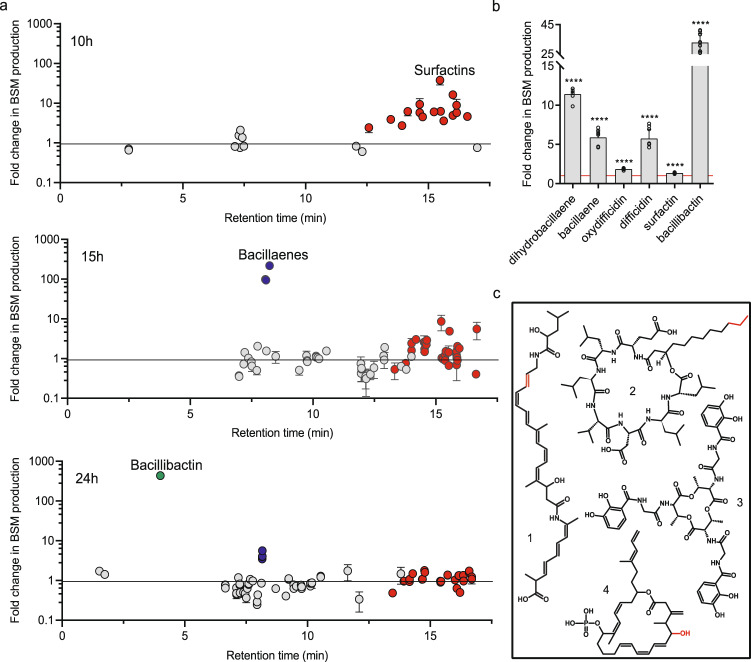
Effect of *P. sessilinigenes* CMR12a supernatant on *B. velezensis* GA1 metabolite production. **a** Impact of CMR12a cell-free supernatant supplementation on the metabolome of GA1 at early exponential phase (10 h), late exponential phase (15 h) and stationary phase (24 h). Each dot represents a feature detected. The dots colored in red, blue, and green represent the features (variants or adducts) of surfactins, bacillaene, and bacillibactin, respectively while gray dots represent the rest of GA1 metabolites. Overproduction data are expressed as peak area fold change per OD600, compared to control culture (un-supplemented GA1 culture). Error bars represent standard deviation (*n* = 3). **b** Fold increase in GA1 bioactive secondary metabolite production upon addition of CMR12a cell-free supernatant (2% (v/v)) compared to un-supplemented cultures (fold change = 1, red line). Data are calculated as indicated in **a**. Mean values were calculated from data obtained in three repeats from three independent experiments (*n* = 9). Statistical significance was calculated using Mann–Whitney test where “****” represents a significant difference at *p* < 0.0001. **c** Overproduced BSMs structures with variable parts in red, explaining the natural co-production of variants for most of the compounds (1: (dihydro)bacillaene, 2: surfactins; 3: bacillibactin; 4: (oxy)difficidin).

For targeted and untargeted analysis of bioactive secondary metabolites, GA1 and CMR12a were cultured in root exudate-mimicking medium or in casamino acids medium, as described above, and on tomato plantlet roots (see the section ‘’*in planta* competition” below). Untargeted analyses of metabolites produced by GA1 (presented on Figs. 1a, S1, S2, S4, and S11) and CMR12a were performed using UPLC MS (Agilent 1290 Infinity II) coupled Jet Stream ESI‐Q‐TOF 6530 mass spectrometer used in positive mode with the parameters described in Supplementary Methods. Data were additionally processed with Mzmine 2 [37] as explained in Supplementary Methods. Targeted analysis of metabolites produced by GA1 (presented on Figs. 1b, 4, S5, and S12) were made by UPLC MS with UPLC (Acquity H-class, Waters) coupled to a single quadrupole mass spectrometer (SQD mass analyzer, Waters), using a C18 column (Acquity UPLC BEH C18 2.1 mm × 50 mm, 1.7 µm), in negative and positive mode. The parameters used for the UPLC MS analysis are described in detail in Supplementary Methods.

### Bioguided fractionation

CMR12a cell-free supernatant was concentrated with a C18 cartridge ‘Chromafix, small’ (Macherey-Nagel, Düren, Germany). The column was conditioned with 10 mL of MeOH followed by 10 mL of milliQ water. Then, 20 mL of supernatant flowed through the column. The metabolites were eluted with 1 mL of a solution of increasing acetonitrile/water ratio from 5:95 to 100:0 (v/v). The triggering effect of these fractions on GA1 PKs production was tested as previously described in the section ‘’Dual interactions”.

### Purification of pyoverdine, E-PCH and PCH

Pyoverdine and E-PCH or PCH, produced by CMR12a or *Pseudomonas aeruginosa* PAO1, respectively, were purified in two steps. Firstly, *Pseudomonas* cell-free supernatant was concentrated with a C18 cartridge (as indicated in section Bioguided fractionation) and eluted with two times 2 mL of a solution of water and acetonitrile (15 and 30% of acetonitrile (v/v)). Secondly, the fractions were injected on HPLC for purification performed on an Eclipse + C18 column (*L* = 150 mm, *D* = 3.0 mm, Particles diameter 5 µm) (Agilent, Waldbronn, Germany). HPLC parameters, pyoverdine, E-PCH, and PCH concentration estimation method used in this study are described in detail in Supplementary Methods.

### *In planta* competition

For *in planta* studies, tomato seeds (*Solanum lycopersicum* var. Moneymaker) were sterilized, deposited on square Petri dishes (5 seeds/plate) containing Hoagland solid medium and placed in the dark for three days [35]. Afterward, 10 seeds were inoculated with 2 µL of the culture (OD_600_ = 0.1) of the appropriate strains (mono-inoculation) or with a mix of GA1 and CMR12a cells (95:5 ration) (co-inoculation) and grown at 22 °C under a 16/8 h night/day cycle with constant light for three days. Inoculum thus corresponds to 1.3 × 10^6^ CFU/plant for GA1 and 2.2 × 10^3^ CFU/plant for CMR12a. To determine colonization rates, root material was collected, and bacteria were detached and homogenized by vortexing for 1 min in peptone water solution supplemented with 0.1% (v/v) of Tween 80. Serial dilutions were prepared and 200 µL of each were plated onto solid LB medium using glass plating beads. After 24 h of incubation at 30 °C for CMR12a and at 42 °C for GA1, colonies were counted. The colonization results were log-transformed and further statistically analyzed. In addition, detached bacteria were used to investigate *acnA*, *srfaA*, *dfnA*, and *baeJ* gene expression by RT-qPCR, as described in Supplementary material.

For GA1 and CMR12a bioactive secondary metabolites production analysis in *in planta* conditions, plants were inoculated and incubated as indicated above for 7 days. An agar part (1 × 2.5 cm) near the tomato roots was cut and bioactive secondary metabolites were extracted with 1.5 mL of acetonitrile (85% (v/v)) for 15 min at room temperature (approx. 22 °C). Further, the samples were centrifuged for 5 min at 4000 rpm and the supernatant was collected for UPLC-MS analysis as described above. Four independent assays each involving at least four plants per treatment, were performed.

### Distribution of sequence homologs of the PchE protein in bacterial genera

To look for sequence homologs of the amino-acid sequence of the PchE protein, we constructed a database of BLASTp results (*n* = 1000). Sequences extracted from the database were clustered at 95% identity using the program CD-HIT [38] and representative sequences were selected for each cluster to remove redundancy (*n* = 289). We further filtered the selected sequences to share >40% identity with the original PchE protein sequence from *P. sessilinigenes* and cover >90% of the BLAST query (*n* = 253). A ClustalW multiple sequence alignment was performed on the resulting sequences with the R package *msa* [39]. Distance matrix and construction of the Neighbor-Joining phylogenetic tree were constructed with the R package *ape* [40]. Visualization of the phylogenetic tree was performed on the iTol web server [41]. For clarity, genera represented by only one sequence were discarded in the final tree.

### Statistical analysis

For statistical analyses, software GraphPad PRISM 8 with Mann–Whitney or Student paired *T*-test was performed. Further, the RStudio 1.1.423 statistical software environment (R language version 4.03) [42]was used for multiple comparisons where one-way ANOVA and Tukey’s Honest Significant Distance (HSD) tests were performed. The groups that differed significantly from each other, at *α* = 0.05, were labeled with different letters.

## Results

### *B. velezensis* modulates its secondary metabolome upon sensing *Pseudomonas* metabolites

We used the genetically amenable, natural rhizosphere isolate GA1 as representative of the *B. velezensis* species. Indeed, based on genome mining, it retains the potential to produce the whole panoply of bioactive secondary metabolites typically formed by members of this species [36, 43]. This is confirmed by efficient production of the whole range of metabolites upon growth in a so-called exudate-mimicking medium, reflecting the content in major carbon sources released by roots of *Solanaceae* plants [34] (Fig. S1). It includes the three families of CLPs (surfactin, iturin, and fengycin), the dipeptide bacylisin, the siderophore bacillibactin and three families of polyketides (bacillaene, difficidin, and macrolactin) [36, 43]. In addition, genes encoding ribosomally synthesized and post-translationally modified peptides such as amylocyclicin and amylolysin are also present in the GA1 genome [36]. Still, these compounds could not be reliably detected in culture broths based on the accurate mass determined via UPLC-qTOF MS. We selected as main interaction partner the plant-associated isolate *P. sessilinigenes* CMR12a based on its production of multiple secondary metabolites such as the siderophores pyoverdin and enantio-pyochelin (E-PCH), the antibiotic phenazine as well as two structurally distinct CLPs, sessilins and orfamides [29–32, 36]. These compounds were identified in culture broth upon growth in casamino acids medium commonly used for *Pseudomonas* cultivation in iron-limited conditions (Fig. S2).

We evaluated the potential of GA1 to modulate secondary metabolite production upon interaction with CMR12a under conditions avoiding possible interferences due to diffusion constraints in a semi-solid matrix or due to the formation of impermeable biofilm structures. Assays were thus performed by growing GA1 in agitated liquid medium supplemented or not with *Pseudomonas* cell-free supernatant (referred here below as crude extract, 2% (v/v)) containing metabolites produced by CMR12a in casamino acids medium. We first employed an untargeted UPLC-MS approach to compare metabolites secreted by GA1 in the two conditions in samples collected at three different time points corresponding to the beginning and the end of the exponential phase or the stationary phase. Data processing with MZmine 2 allowed us to detect features and the corresponding natural products differentially produced upon interaction. It revealed a growth phase-dependent differential accumulation of three secondary metabolites upon supplementation with *Pseudomonas* crude extract (Figs. 1a, Fig. S3 for GA1 growth kinetics). The production of surfactins was strongly increased in the early exponential phase, bacillaene or its dehydrated variant dihydrobacillaene (2H-bae) was stimulated at the transition from exponential to stationary phase and synthesis of the siderophore bacillibactin was enhanced in the stationary phase (Fig. 1a). The amplitude and timing of the boosting effect thus varied according to the molecule, most probably due to specific transcriptional regulations. The production of other compounds such as fengycins, iturins, macrolactins, and bacilysin was not impacted by the addition of CMR12a crude extract (Fig. S4). We next set out to inspect the whole range of natural products after 20 h of growth using an optimized UPLC-MS method for targeting non-ribosomal compounds. It confirmed the differential accumulation of (dihydro)bacillaene, surfactins, and bacillibactin but also revealed a significantly enhanced production of difficidin or its oxidized form (only detected in ESI negative mode) upon the addition of CMR12a extract (Fig. 1b, chemical structures in Fig. 1c). We observed a globally similar impact of the *Pseudomonas* CMR12a extract on secondary metabolite production by other *B. velezensis* biocontrol strains such as S499, FZB42, and QST713 (Fig. S5) [44, 45], indicating that such interaction-mediated metabolite response is conserved among members of the species.

### Bioactive secondary metabolite stimulation leads to enhanced antibacterial potential

**Fig. 2:**
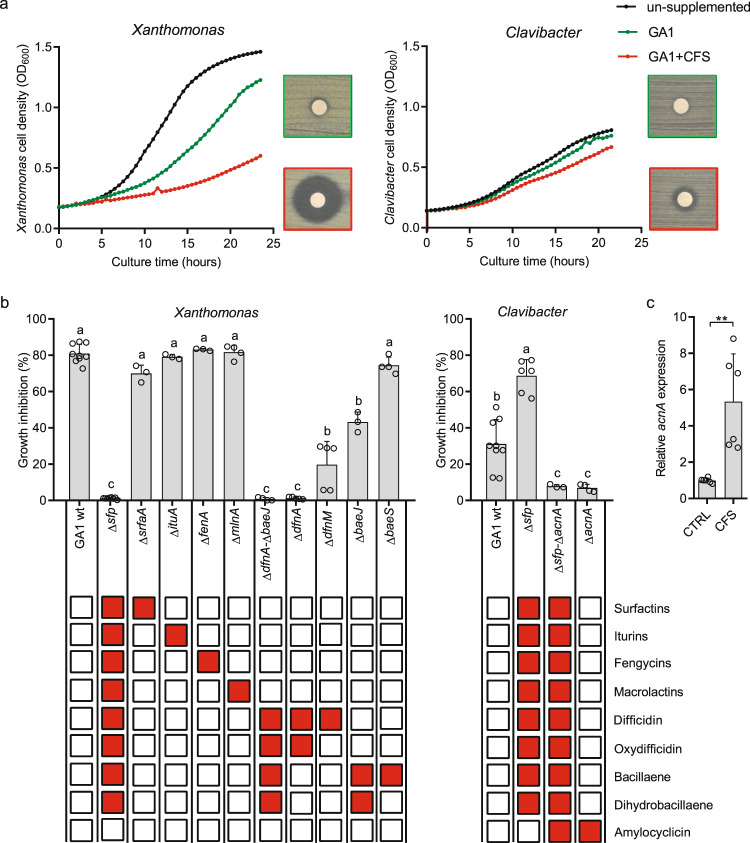
*B. velezensis* GA1 antibacterial activities are enhanced in response to *P. sessilinigenes* CMR12a secreted metabolites and rely on the production of different bioactive secondary metabolites according to the target species. **a** The enhanced anti-*Xanthomonas campestris* and anti-*Clavibacter michiganensis* activities of GA1 cell-free culture supernatants (CFS) after growth in CMR12a CFS-supplemented medium (GA1 + CFS) compared to un-supplemented control (GA1). It was assessed both on plates by the increase in inhibition zone around paper discs soaked with GA1 extracts and in liquid cultures of the pathogens by reduction of growth upon addition of GA1 extracts. Data are from one representative repetition. **b** Antibacterial activities of extracts from GA1 wild-type (GA1 wt) and mutants in in production of specific compounds without supplementation with CMR12a cell-free supernatant. Metabolites not produced by the different mutants are illustrated with red boxes in the table below. All values represent means with error bars indicating ± SD calculated on data from three cultures (repeats) in two independent experiments (*n* = 6) and 0% represents a total loss of the strain activity while 100% represents total retention of strain activity (total inhibition of the pathogen). Letters a to d indicate statistically significant differences according to one-way analysis of variance (ANOVA) and Tukey’s HSD test (Honestly significantly different, *α* = 0.05). **c** Differential expression of the *acnA* gene encoding the amylocylicin precursor, upon supplementation with CMR12a CFS compared to GA1 un-supplemented culture. Mean and ± SD values are recalculated from three repetitions from two biological replicates (*n* = 6) where “**” indicates statistical significance according to Mann–Whitney test, *p* < 0.01.

PKs (i.e. (dihydro)bacillaene, (oxy)difficidin, and macrolactins) are among the secondary metabolites of *Bacillus* best described for their inhibitory activity toward a wide range of bacteria [15, 16]. We thus speculated that an enhanced antibacterial activity would be a direct outcome of this GA1 metabolite response to CMR12a products. This was observed by testing *Bacillus* extracts resulting from the interaction, for growth inhibition of *Xanthomonas campestris* and *Clavibacter michiganensis* used respectively as representative of Gram-negative and Gram-positive plant pathogenic bacteria of agronomical importance [46] (Fig. 2a). In these conditions, no effect of the *Pseudomonas* cell-free supernatant (representing 0.08% (v/v) (0.04*0.02) was observed on pathogen growth, obviously due to the very low concentration of metabolites in the medium. To determine the specific involvement of each metabolite in bacterial inhibition, we generated and tested a range of GA1 knock-out mutants, including the Δ*sfp* derivative specifically repressed in 4′-phosphopantetheinyl transferase, which is essential for the proper functioning of the PK and non-ribosomal peptide biosynthesis machineries (i.e. responsible for the synthesis of PKs, CLPs, and bacillibactin). Total loss of anti-*Xanthomonas* activity in Δ*sfp* extracts indicated a key role for those compounds (Fig. 2b) and ruled out the possible involvement of other molecules known for their antibacterial activity, such as bacilysin or ribosomally synthesized and post-translationally modified peptides (Fig. S6a). Loss of function of mutants specifically repressed in the synthesis of individual compounds pointed out the key role of (oxy)difficidin and, to a lower extent, (dihydro)bacillaene in *X. campestris* inhibition (Fig. 2b). Acting alone or in synergy, these two PKs are also responsible for GA1 inhibitory activity toward other important bacterial phytopathogens such as *P. carotovorum, A. tumefaciens*, and *R. fasciens* but are not involved in the inhibition of the plant pathogenic *P. fuscovaginae* species for which bacilysin may be the active metabolite (Fig. S6b). However, GA1 does not display significant toxicity against the non-pathogenic soil *P. sessilinigenes* CMR12a and *P. protegens* Pf-5 tested here (Fig. S7).

In contrast to *X. campestris*, enhanced antibiotic activity against *C. michiganensis* is not mediated by non-ribosomal products as shown by the high activity in the Δ*sfp* mutant (Fig. 2b). The activity of the *Δsfp* mutant against *C. michiganensis* was even significantly increased compared to GA1 wild type. In this mutant, production of the otherwise efficiently produced CLPs is abolished. Building on this, we expect that amino acids required for CLPs synthesis to be spared and we hypothesized that they might be redirected to the production of ribosomal peptides, including the ones active against *C. michiganensis*, leading to an enhanced activity. Therefore, we suspected from genomic data and literature [47] that ribosomally synthesized and post-translationally modified peptides, such as amylocyclicin could be involved in inhibition. This hypothesis was supported by the drastic loss in pathogen inhibition potential observed for the Δ*acnA* mutant knocked-out for the corresponding biosynthesis gene (Fig. 2b). Moreover, mutants of another antimicrobial peptide amylolysin, and the *sfp*-independent, non-ribosomal peptide bacilysin did not lose their activity (Fig. S6a), pointing out amylocyclicin as the main metabolite antagonizing *Clavibacter*. We were not able to provide evidence for higher accumulation of the mature peptide in the medium, but RT-qPCR data revealed a strongly induced expression of *acnA* gene in GA1 cells in response to CMR12a (Fig. 2c). Based on genome mining, S499 is another strain of the *B. velezensis* species that also retains the potential to produce amylocyclicin [44] and enhanced expression of the *acnA* gene in the presence of CMR12a products was also observed with this isolate (Fig. S8).

### Secondary metabolite synthesis is enhanced upon competitive root colonization

**Fig. 3:**
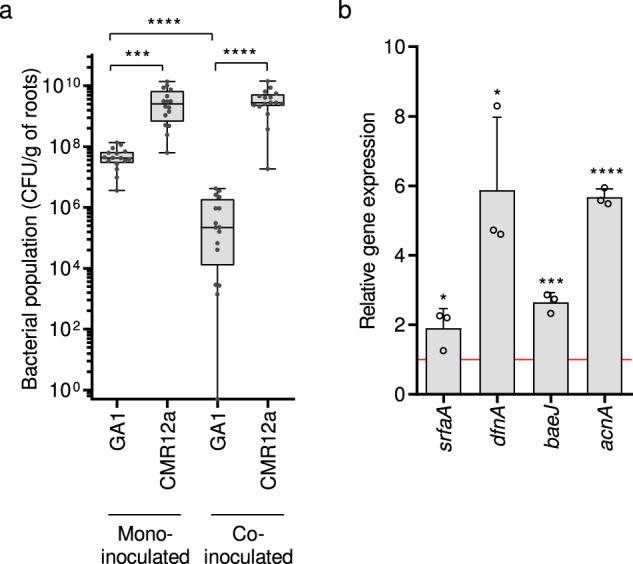
Competitive colonization assays support the roles of bioactive secondary metabolites in *Bacillus-Pseudomonas* interaction *in planta*. **a** GA1 and CMR12a cell populations as recovered from roots at 3 days post-inoculation (dpi) of tomato plantlets when mono- or co-inoculated. Box plots were created from data from four independent assays each involving at least 4 plants per treatment (*n* = 16). The whiskers encompass the minimum and maximum values, and the midline shows the median. Statistical differences between the treatments were analyzed using Mann–Whitney test and ‘’****” and ‘’***” represent significant differences at *p* < 0.0001 and *p* < 0.001, respectively. **b** *In planta* (3 dpi on tomato roots) GA1 relative expression of the *sfraA*, *dfnA*, *baeJ*, and *acnA* genes responsible for the synthesis of surfactins, (oxy)difficidin, (dihydro-)bacillaene, and amylocyclicin, respectively. Graphs show the mean and ± SD calculated from three biological replicates (*n* = 3), each involving six plants. Fold change = 1 as red line corresponds to gene expressions in GA1 inoculated alone on roots, used as control conditions. Statistical comparison between data in co-colonization setting and control conditions was performed based on *T*-test (*p < 0.05; ***p < 0.001; ****p < 0.0001).

These in vitro data thus illustrate how *B. velezensis* modulates its secondary metabolome in response to some *Pseudomonas* compounds secreted by planktonic cells grown in casamino acids medium. However, except for pyoverdine, all CMR12a metabolites are readily produced in the exudate-mimicking medium and substantial amounts are also formed when the bacterium colonizes tomato roots (Figs. S1 and S2). Therefore, we next performed *in planta* experiments with tomato plantlets grown at 22 °C [48] in order to test metabolite stimulation in GA1 upon root co-colonization in presence of CMR12a. Both isolates efficiently colonized roots when inoculated individually but upon competitive root invasion, CMR12a overgrows GA1, which forms significantly lower populations compared to mono-inoculated plantlets (Fig. 3a). This may be explained by a higher intrinsic growth rate and/or better use of exudates by *Pseudomonas*, but also by the production of antimicrobials like the lipopeptide sessilin recently described for its inhibitory activity toward *B. velezensis* [36]. Due to these reduced populations, we could not reliably detect all GA1 metabolites in rhizosphere extracts from co-inoculated plants. However, a significantly enhanced expression of gene clusters responsible for the synthesis of surfactin, (dihydro)bacillaene, (oxy)difficidin, and amylocyclicin was observed in GA1 cells co-inoculated with CMR12a compared to single inoculation (Fig. 3b). It indicates that metabolite stimulation observed for planktonic GA1 cells also occurs in a context of competitive colonization where the bacterium forms biofilm, feeds exclusively on root exudates and is obviously in close contact with colonies of the challenger [36, 49, 50].

### Pyochelin acts as *Pseudomonas* signal sensed by *Bacillus*

We next wanted to further investigate the molecular basis of this chemical dialogue and identify the compound(s) secreted by CMR12a that is(are) sensed by *Bacillus* cells to trigger the production of antimicrobials. For that purpose, we used 2H-bae as the main indicator of the *Bacillus* response because it represents the most highly boosted metabolite with antibacterial activity considering that a direct toxic activity for bacillibactin has not been reported so far.

**Fig. 4:**
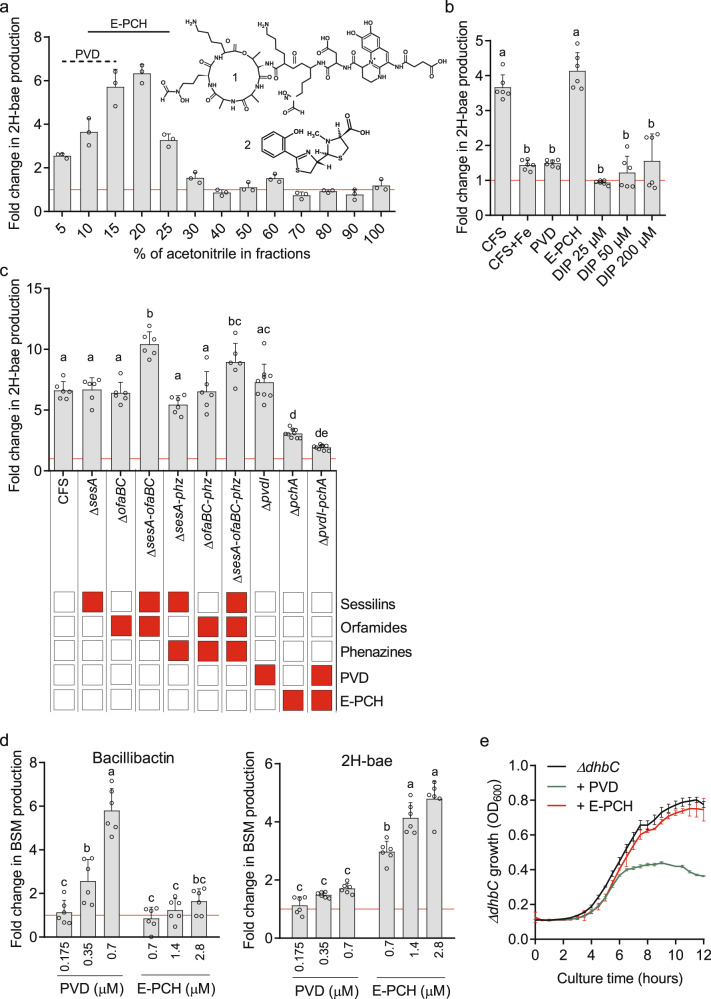
Enantio-pyochelin as main *P. sessilinigenes* CMR12a trigger of secondary metabolite boost in *B. velezensis* GA1. **a** The CMR12a cell-free supernatant (CFS) was processed through the solid-phase extraction on C18 cartridges and compounds were stepwise eluted according to their hydrophobicity with increasing acetonitrile-water ratio, expressed in ACN % (v/v). All fractions were analyzed by UPLC-MS and those containing substantial amounts of pyoverdine (PVD) (1: PVD structure) and/or enantio-pyochelin (E-PCH) (2: E-PCH structure), labeled as dashed and solid lines, respectively. Fold changes were calculated based on relative quantification of the compounds by UPLC-MS (peak area) per OD600 in treated cultures compared to un-supplemented controls (fold change = 1, red line). Data are from one representative experiment showing mean and ± SD calculated from three technical replicates. **b** Differential production of dihydrobacillaene (2H-bae) after addition of 0.35 µM pure PVD, 1.4 µM pure E-PCH, 2% (v/v) CMR12a cell-free supernatant (CFS), CMR12a CFS from iron supplemented culture (CFS + Fe) and different concentration of the iron-chelating agent 2,2′-dipyridyl (DIP). Fold changes were calculated as indicated in **a**. **c** Effect of GA1 culture supplementation with CFS (2% (v/v)) from CMR12a wild type and various mutants on 2H-bae production. Metabolites specifically repressed in the CMR12a mutants are illustrated by red boxes. Fold changes were calculated as indicated in **a**. **d** Dose-dependent effect of pure PVD and E-PCH on bacillibactin and 2H-bae production. GA1 cultures were supplemented with the indicated concentrations of HPLC-purified CMR12a siderophores. Fold changes were calculated as indicated in **a**. Data represented on **b**, **c**, and **d** are means and ±SD calculated from three replicate cultures in two independent experiments (*n* = 6) where different letters indicate statistically significant differences (ANOVA and Tukey’s HSD test, *α* = 0.05) for 2H-bae and bacillibactin, the control (i.e. un-supplemented GA1 culture) belonged to the c group. **e** Impact of the addition of pure PVD and E-PCH on the growth of GA1 Δ*dhbC* mutant repressed in bacillibactin synthesis. CMR12a siderophores were added at a final concentration similar to the one obtained by adding CMR12a CFS at 2% (v/v) (0.35 µM of PVD and 1.4 µM of E-PCH). Means and ± SD are from three replicates. For detailed statistical analysis, see Fig. S10.

We first performed bioactivity-guided fractionation of the CMR12a cell-free supernatant on C18 solid-phase extraction cartridges. It revealed that only extracts containing pyoverdine and/or E-PCH displayed consistent PK-triggering activity (Fig. 4a). This possible involvement of siderophores was supported by the drastic reduction in the activity of cell-free supernatant prepared from CMR12a culture in casamino acids medium supplemented with Fe^3+^ where their production is repressed (Figs. 4b and S9). We next compared the triggering potential of cell-free supernatant obtained from cultures of CMR12a knock-out mutants specifically lacking the different identified metabolites but not being disrupted in the production of other metabolites [51]. As expected, only extracts from mutants impaired in the production of E-PCH were significantly affected in PK-inducing potential (Fig. 4c). To confirm this differential effect of the two siderophores, we then tested the HPLC-purified compounds independently at a concentration similar to the one measured in *Pseudomonas* cell-free supernatant. Data showed a much higher PK-triggering activity for E-PCH compared to the main pyoverdine isoform produced by CMR12a (Fig. 4b).

In additional dose-dependent assays, we observed that supplementation with pyoverdine caused iron limitation in the medium, which is sensed by GA1. This is illustrated by the marked increase in production of the siderophore bacillibactin in GA1 wild-type (Fig. 4d) and by the reduced growth of the Δ*dhbC* mutant, repressed in bacillibactin synthesis, upon pyoverdine addition (Figs. 4e and S10). Our genome inspection revealed that *B. velezensis* GA1 has acquired multiple transporters allowing the uptake of exogenous siderophores (Table S3) similar to those identified in *B. subtilis* [52, 53]. However, data indicate that pyoverdine (in its ferric form) cannot be taken up by GA1. As strong chelator, pyoverdine scavenges most of the available iron in the external medium and causes iron stress in GA1 cells. This leads to an enhanced production of the GA1 siderophore bacillibactin in a process mediated by the transcriptional repressor Fur [54]. The addition of pyoverdine or the 2,2′-dipyridyl (DIP) chemical chelator, both not internalized and not used by *Bacillus* cells [54], also leads to a slight but not significant stimulation of 2H-bae synthesis (Fig. 4b, d). We assume that such low 2H-bae increase is part of a global response to iron limitation caused by the presence of exogenous siderophores/chelators as previously reported [52, 53]. In support, the synthesis of 2H-bae in GA1 is under the control of the global regulator Fur involved in iron homeostasis and controlling bacillibactin production as *de novo* synthesis of 2H-bae is significantly enhanced in the Fur-depleted mutant (Fig. S11a). This regulation is most probably indirect since we could not identify any Fur binding site (Fur-box) in the promoter region of the *bae* operon (Table S4).

By contrast to pyoverdine, the addition of the secondary siderophore E-PCH with a much lower affinity for iron strongly stimulates 2H-bae production but does not activate bacillibactin synthesis (Fig. 4d) and does not affect Δ*dhbC* growth at the concentrations used (Figs. 4e and S10). Therefore, E-PCH does not act on GA1 cells by causing iron limitation as further supported by the fact that stimulation of 2H-bae synthesis by E-PCH is conserved in the Δ*furR* mutant with no impact on growth (Fig. S11a, b. That said, E-PCH was described to retain some antibiotic activity by causing oxidative stress and damage as reported in *Escherichia coli* [55, 56]. However, E-PCH does not display any toxicity toward GA1 wild-type and the Δ*dhbC* mutant nor markedly affects the growth of the *ΔperR* mutant repressed in the major regulator involved in *Bacillus* oxidative stress response (Fig. S11b) [57]. Moreover, a consistent boost in 2H-bae is also conserved in this *ΔperR* mutant (Fig. S11a), further indicating that E-PCH does not induce oxidative stress by acting intracellularly and thereby supporting its perception as exogenous info-chemical by *Bacillus* cells. Such signaling activity is not impacted by the stereochemistry of the molecule since we observed a similar increase in polyketide production (6.05 ± 1.8-fold-increase, *n* = 4) by testing the other natural isomer pyochelin (PCH) purified from *P. aeruginosa* PAO1 [58].

*P. protegens* strains such as Pf-5 and CHA0 have also been described as E-PCH producers [59]. We also observed a boost in PK production similar to CMR12a upon adding cell-free supernantant prepared from Pf-5 (Fig. S12). The *pchE* gene encodes key enzymes forming the 2-hydroxyphenyl-thiazolinyl-thioester intermediate of the linear tricyclic PCH scaffold, which are very similar (>92% identity) in the taxonomically closely related species *P. protegens* and *P. sessilinigenes* [59] (Fig. S13). In the literature, production of PCH or structurally related compounds has also been described for *Burkholderia* spp. and *Streptomyces* spp [60–62]. but PchE sequences are much less conserved (<50% identity, Fig. S13), suggesting that these proteins have a similar function but have probably evolved independently.

**Fig. 5:**
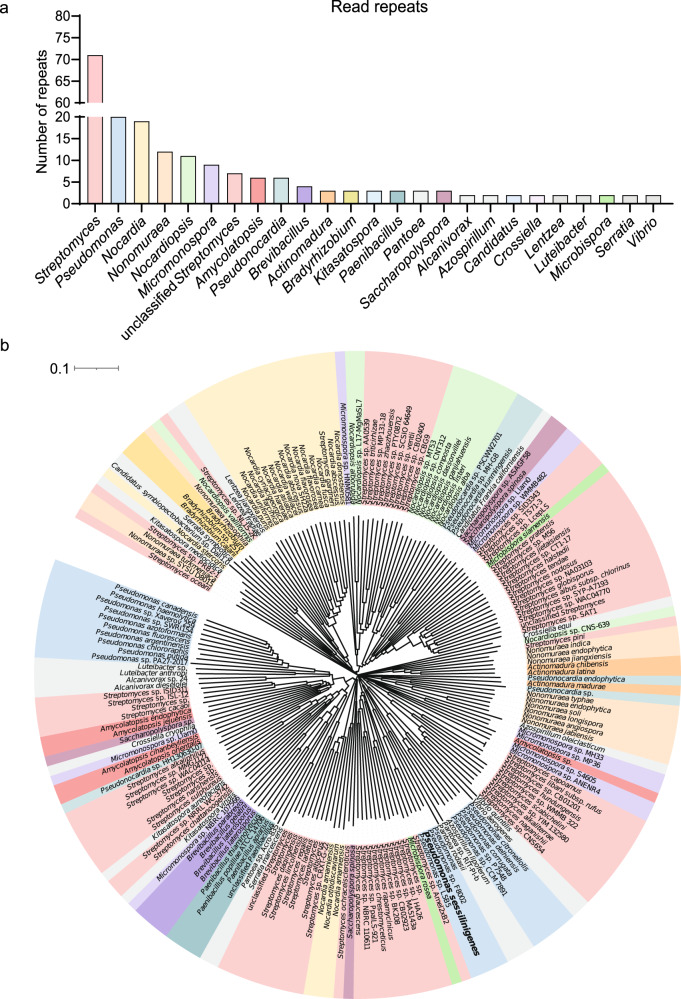
Distribution of sequence homologs of the PchE protein in diverse bacterial genera. **a** Frequency of each genus that expresses close homologs of the PchE protein, expressed as several individual representative isolates (y-axis) within the specific genus (x-axis) following filtering of BLASTp results (*n* = 253). **b** Neighbor-Joining phylogenetic tree of bacterial isolates containing putative homologs of the PchE protein. For clarity purposes, genera represented by only one sequence were removed from the tree. Distribution of bacterial clades such as *Streptomyces*, *Nocardia*, or *Pseudomonas* across the tree could suggest the presence of both paralog and ortholog genes encoding PchE-like proteins in different species of the same genus.

We also wanted to search across bacterial phyla for putative homologs of the PchE protein encoded by *pchE* gene in order to evaluate how widespread the potential to produce PCH can be. In support to the importance of this gene and associated compound, it first revealed a high conservation (>40% sequence identity) over almost the entire sequence (>90%) in phylogenetically distant clades of bacteria. After filtering of the BLAST results, putative homologs of the PchE protein were found to be present in a wide diversity of bacteria, including many pseudomonads and actinomycetes such as *Streptomyces*, *Nocardia* and *Micromonospora* (Fig. 5a). In support to these data, production of PCH or PCH homologs has been reported for these actinomycetes species and for members of the *Brevibacillus* and *Vibrio* genera [61, 63–65]. All the species identified have been described as soil-dwelling bacteria that can thus be found in the same ecological niche as *B. velezensis* [66–74]. Next, ClustalW multiple sequence alignment performed on 253 representative sequences based on blast results allowed building a Neighbor-Joining phylogenetic tree (Fig. 5b). We observed the clustering of the same genera in several subdivisions of the tree, which potentially indicates the presence of close paralogs of genes encoding PchE-related proteins, mainly among *Streptomyces* species. This suggests some diversity in the non-ribosomal peptide synthetase machinery and in the structure of the PCH-related synthesized compounds.

## Discussion

In this study, we investigated the interaction between *Bacillus* and *Pseudomonas* as bacterial genera predominant in rhizosphere microbiomes and with strong biocontrol potential to protect plants against diseases. We show for the first time that *B. velezensis* can mobilize a substantial part of its bioactive secondary metabolome in response to the perception of a bacterial competitor and more specifically upon sensing pyochelin formed by *Pseudomonas*. The stimulated metabolites include the bacillaene and difficidin-type polyketides as well as their hydroxy- and oxy- derivatives well described for their broad-spectrum antibiotic activities against Gram-negative, together with the bacteriocin amylocyclicin with strong antagonistic potential mostly against Gram-positive and related species [16, 47]. Enhanced production of these compounds would thus provide a clear benefit as it improves the potential of *B. velezensis* to fight a range of bacterial competitors. Due to its bacteriostatic activity, bacillaene is also viewed as defence chemical in antagonistic interactions as it protects *Bacillus* from predation by *Myxococcus xanthus* [75] and plays an essential protective role for survival in competition with *Streptomyces* soil isolates [76, 77] and in the interaction with *Serratia plymuthica* [78]. Interestingly, the bacteriostatic effect of bacillaene also protects *B. velezensis* strain FZB42 toward colony invasion by *Pseudomonas chlororaphis* [24]. According to our results, bacillaene (or its hydroxy- form) is not toxic for the *P. sessilinigenes* and *P. protegens* challengers tested but can thus be inhibitory for other *Pseudomonas* species like *P. chlororaphis*. In addition, our findings can explain the higher amounts of bacillaene that accumulate upon interaction between FZB42 and *P. chlororaphis* as previously reported [24] since the response to pyochelin observed for GA1 is conserved in strain FZB42 (Fig. S5) and since the *P. chlororaphis* strain used in that work forms pyochelin. Therefore, metabolite stimulation by *Bacillus* should be viewed as a response to pyochelin as particular molecular cue, whatever the nature and level of sensitivity of the species releasing this signal (see also below). It is worth noticing that secondary metabolites mainly described for their antifungal properties such as iturin or fengycin are not stimulated upon interaction with *Pseudomonas* while the production of these lipopeptides is increased in interkingdom interactions with fungi and oomycetes [22]. *B. velezensis* would thus raise the proper subset of its chemical weapon arsenal according to the nature of the microbial challenger.

Surfactin production is also stimulated by PCH but a direct role of this CLP as antibiotic in antagonistic interactions is obviously minor. Surfactin can interfere with the growth of closely related species in synergy with cannibalism toxins [79] or inhibit the development of *Streptomyces* aerial hyphae [80, 81]. However, these effects are not directly correlated with antibiotic activity and, more globally, toxicity of this CLP toward soil-dwelling microbes has not yet been convincingly reported at biologically relevant concentrations [82, 83]. Still, surfactin is important for *Bacillus* fitness in the rhizosphere since it favors key developmental processes such as motility and biofilm formation [22]. Enhanced multicellular motility in *B. subtilis* as a response to the perception of exogenous compounds has been demonstrated and can be viewed as an escape mechanism used after detecting harmful challengers [82, 84]. Biofilm formation on the root surface is a natural trait of *Bacillus* and boosting this surfactin-dependent process in response to external signals may also represent a way to protect the community against toxins and/or infiltration by competitors via the shield effect of the hydrophobin layer [25, 36, 85, 86]. In interaction with CMR12a, surfactin protects *B. velezensis* against the sessilin-mediated toxicity of *Pseudomonas* via co-aggregation with this large lipopeptide leading to the formation of insoluble supra-molecular complexes [36]. With this protective effect, surfactin somehow contributes to the *Bacillus* competitiveness for colonization even if GA1 is overgrown by CMR12a upon co-inoculation on roots. Some *Pseudomonas* isolates may inhibit *Bacillus* via antibiotics or toxins delivered by type VI secretion system [25] but this is not a general rule. Many species do not interfere with *Bacillus* development and form stable mixed biofilms in vitro and mixed communities on roots where overgrowth is not observed [36, 87]. Instead of developing sophisticated competition strategies, some species of the two genera may establish syntrophic interactions based on metabolic cross-feeding and exchange of nutrients [87]. In addition to antibacterial polyketides and bacteriocin, the stimulation of surfactin production may thus also indirectly provide benefits to *Bacillus* in terms of protection against harmful species. Therefore, we assume that, together with other traits such as sporulation and genetic adaptation to antibiotic-mediated inhibition [24], the *Bacillus* metabolic response reported here and involving various compounds with specific functions, contributes to the development of a multi-faceted strategy to increase fitness and ensure persistence in its natural niche.

With this work, we also unveil a new communication system featuring the *Pseudomonas* compound PCH as info-chemical sensed by *B. velezensis* to boost its chemical arsenal. This points out a new role in interspecies interactions for PCH-type metabolites beyond their prime function as a secondary siderophore with lower affinity than the main chelator (pyoverdine) but with specific regulation allowing to adapt more flexibly their metal acquisition system to external conditions [88]. Mechanistically, *Bacillus* perceives PCH independently of iron stress and siderophore piracy, unlike siderophore-mediated interaction described so far [9]. In the pairwise system used here, E-PCH signaling indeed superimposes the possible effect of iron limitation in the external medium which may also result in enhanced production of antibacterial metabolites by *Bacillus*, as occasionally reported in other species [89, 90].

Due to the limitation in bioavailable iron, almost all known rhizobacterial species have adapted to produce their own iron-scavenging molecules in order to compete efficiently for this essential element [90, 91]. Siderophore production is thus widely conserved among soil-borne bacteria [90]. It means that upon recognition of exogenous siderophores, any isolate may somehow identify surrounding competitors. However, some of these siderophores such as pyoverdines from fluorescent pseudomonads, are structurally very variable and almost strain-specific while others like enterobactin or citrate are much more widely distributed across species and even genera [90]. In both cases, their recognition would not provide proper information about the producer because they are respectively too specific with a perception at the level of the strain but blind to closely related competitors, or too general for allowing proper discrimination. Results from our genomic survey illustrate an unsuspected widespread potential for the synthesis of PCH or structurally close derivatives among soil-dwelling species. Therefore, we hypothesize that *Bacillus* may have evolved some sensing systems targeting siderophores that are conserved enough to be detected as relevant info-chemical but, in a delicate balance, also restricted to specific microbial phylogenetic groups able to produce PCH or variants [92]. With this mechanism, soil bacilli would rely on specific exogenous siderophores as an “eavesdropping” strategy to accurately identify competitors and respond appropriately by remodeling the expression of the secondary metabolome. This novel concept of chelator sensing thus represents a new facet of siderophore-mediated interactions beyond competition for iron. Nevertheless, soil pseudomonads produce other secondary siderophores than PCH being salicyl-capped derivatives or structurally unrelated compounds such as achromobactin and pseudomonine [92, 93]. These compounds should also be tested for their activity as triggers of PKs synthesis in *Bacillus* to evaluate whether the chelator-sensing concept can be broadened to a larger panel of exogenous metal scavengers or not. Assessing whether the adaptative response of *B. velezensis* can be generalized to other soil-dwelling species also deserves to be further investigated as it provides novel insights into the ecology of soil bacteria. That said, the mechanism of E-PCH perception remains unrevealed. *B. subtilis* can uptake xenosiderophores which in turn induce sporulation [52]. However, our data suggest that PCH is not internalized based on the behavior of mutants and on the absence of genes putatively encoding proteins with similarity with the well-known PCH transporters in the producing bacteria. Moreover, the presence of exogenous siderophores accelerates the differentiation of *Bacillus* vegetative cells into spores as reported for enterobactin and ferrioxamine [52]. It is explained by the fact that *Bacillus* readily takes up these piratable siderophores which causes some intracellular oxidative stress leading to enhanced sporulation as a response. Such a higher sporulation cannot be suspected from our experiments (data not shown) and PCH from *P. chlororaphis* does not affect *B. subtilis* sporulation rate as previously reported [25]. Although it does not provide conclusive evidence, this further supports the fact that PCH cannot be actively transported by *Bacillus* cells [25]. The involvement of a putative receptor at the cell membrane is being further investigated together with the possible involvement of some global regulators, integrating PCH perception into downstream transcriptional activation of the biosynthetic gene clusters responsible for the synthesis of those costly secondary metabolites.

Here, we report a new role for PCH as signal mediating interspecies interactions. Some implication of PCH (or E-PCH) in microbial interactions has been reported but mainly in an antagonistic context where the siderophore notably acts via iron-depletion in iron-limited conditions to restrict growth of other bacteria, fungal pathogens or ectomycorrhizal fungi [94]. *Staphylococcus aureus* produces an enzyme that methylates the carboxylic acid moiety of PCH, which reduces the efficiency of the PCH-mediated iron-acquisition system and significantly lowers pyochelin-mediated intracellular ROS production and oxidative stress [95]. PCH produced by *Burkholderia cenocepacia* also displays toxicity toward the plant pathogen *Phellinus noxius* but is modified by the fungus to an esterification product with lower affinity for iron and loss of antifungal activity [96]. In another way, the fungus *Candida albicans* also attenuates the pyochelin-mediated virulence of *P. aeruginosa* by inhibiting PCH gene expression [97]. These reports illustrate competition strategies mounted by microbial challengers to counteract PCH inhibitory activity and to co-exist with bacterial producers. However, a role as signal for this secondary siderophore has only been exemplified in interaction with the nematode *Caenorhabditis elegans* where it serves as an environmental cue sensed via the neuroendocrine signaling pathway to promote avoidance of pathogenic *P. aeruginosa* [98].

The discovery of such chelator-sensing phenomenon also further illustrates the diversity of molecular processes driving bacterial interactions in general and those between clades that are important members of the plant-associated microbiome in particular. As it results in an enhanced antimicrobial potential of *Bacillus* without major impact on the growth of both partners, our findings should contribute to rationally design consortia for plant protection. Indeed, multispecies inocula can provide plants with stronger disease resistance and growth promotion than single strains [99, 100]. In particular, several studies have shown the potential of combining *Bacillus* and *Pseudomonas* species [101, 102] but the association of these two strong competitors may also result in antagonistic phenotypes depending on the species tested [25, 103]. A better understanding of the nature and dynamics of interactions between these bacteria is thus still necessary in order to provide a way forward to engineering consortia with predictable compatibility and high biocontrol potential.

## Supplementary information

Supplemental material

## Data Availability

All data needed to evaluate the conclusions in the paper are present in the paper and/or the Supplementary Materials. Additional information related to this article may be demanded from the corresponding authors SA (sofija.andric@uliege.be), TM (thibault.meyer@univ-lyon1.fr) or MO (marc.ongena@uliege.be).
