# Supplementary material for: Plant-associated Bacillus mobilizes its secondary metabolites upon perception of the siderophore pyochelin produced by a Pseudomonas competitor
Source: ISME J. 2022 Nov 10;17(2):263–75. doi: 10.1038/s41396-022-01337-1 (PMC9860033; doi:10.1038/s41396-022-01337-1)
Supplement: Supplementary file 1 — Supplemental material [file 41396_2022_1337_MOESM1_ESM.docx]

ISME J

SUPPLEMENTARY INFORMATION

Plant-associated *Bacillus* mobilizes its secondary metabolites upon perception of the siderophore pyochelin produced by a *Pseudomonas* competitor

Andrić et al.

Includes Supplementary Methods, Supplementary Figures S1-S13, Supplementary Tables S1-S4, and Supplementary References

**SUPPLEMENTARY METHODS**

***Pseudomonas* cell-free supernatant**

*Pseudomonas* cell suspension was adjusted to OD_600nm_ 0.05 in 100 mL of casamino acids medium and, when appropriate, supplemented with 20 µg/L of FeCl_3_x6H_2_O (iron supplementation). Cultures were orbitally shaken at 120 rpm at 30 °C for 48 h. Finally, the cultures were centrifuged at 5000 rpm at room temperature (approx. 22 °C) for 20 min. The supernatants were further filter-sterilized (0.22 µm pore size filters) and stored at -20 °C until further use.

**RNA isolation and RT-qPCR**

To investigate *acnA*, *srfaA*, *dfnA,* and *baeJ* gene expression in *Bacillus*, firstly RNA extraction and DNAse treatment were carried out using the NucleoSpin RNA Kit (Macherey Nagel, Germany), following the Gram + manufacturer’s protocol. RNA quality and quantity were performed with Thermo scientific NanoDrop 2000 UV-vis Spectrophotometer. Primer 3 program available online was used for primer design and primers were synthesized by Eurogentec. The primers used for this purpose are listed in Table S2. The primer efficiency was evaluated and primer pairs showing an efficiency between 90 and 110% in the qPCR analysis were selected. Reverse transcriptase and RT-qPCR reactions were conducted using the Luna Universal One-Step RT-qPCR Kit (New England Biolabs, Ipswich, MA, United States). The reaction was performed with 50 ng of total RNA in a total volume of 20 μL: 10 μL of luna universal reaction mix, 0.8 μL of each primer (10 μM), 5 μL of cDNA (50ng), 1 μL of RT Enzyme MIX, 2.4 µL of Nuclease-free water. The thermal cycling program applied on the ABI StepOne was: 55 °C for 10 min, 95 °C for 1 min, 40 cycles of 95 °C for 10 s, and 60 °C for 1 min, followed by a melting curve analysis performed using the default program of the ABI StepOne qPCR machine (Applied Biosystems). Finally, the real-time PCR amplification was run on the ABI step-one qPCR instrument (Applied Biosystems) with software version 2.3. The relative gene expression analysis was conducted using the 2*ΔCt* method [1] with the *gyrA* gene as a housekeeping gene to normalize mRNA levels between different samples.

**Secondary metabolite analysis**

The parameters for untargeted analyses of metabolites produced by *Pseudomonas* and *Bacillus* bioactive secondary metabolites identification by UPLC-qTOF MS were set up as follows: parameters: capillary voltage: 3.5 kV; nebulizer pressure: 35 psi; drying gas: 8 L/min; drying gas temperature: 300 °C; ﬂow rate of sheath gas: 11 L/min; sheath gas temperature: 350 °C; fragmentor voltage: 175 V; skimmer voltage: 65 V; octopole RF: 750 V. Accurate mass spectra for *Pseudomonas* bioactive secondary metabolites were recorded in the range of m/z = 40-2500. Accurate mass spectra for *Bacillus* bioactive secondary metabolites were recorded in the range of m/z =100–1700. A C18 Acquity UPLC BEH column (2.1 × 50 mm × 1.7 μm; Waters, milford, MA, USA) was used at a ﬂow rate of 0.3 mL/min (for *Pseudomonas* bioactive secondary metabolites) or 0.6 mL/min (for *Bacillus* bioactive secondary metabolites) and a temperature of 40 °C. The injection volume was 20 μL (for *Pseudomonas* bioactive secondary metabolites) or 10 μL (for *Bacillus* bioactive secondary metabolites) and the diode array detector (DAD) scanned a wavelength spectrum between 190 and 600 nm. For *Pseudomonas* bioactive secondary metabolites, a gradient of 0.1% formic acid water (solvent A) and acetonitrile acidified with 0.1% formic acid (solvent B) was used as a mobile phase with a constant flow rate at 0.45 mL/min starting at 10% B and raising to 100% B in 20 min. Solvent B was kept at 100% for 2 min before going back to the initial ratio. For *Bacillus* bioactive secondary metabolites, a gradient of 0.1% formic acid (solvent A) and acetonitrile acidified with 0.1% formic acid (solvent B) was used as a mobile phase with a constant flow rate at 0.6 mL/min starting at 10% B and raising to 100% B in 20 min. Solvent B was kept at 100% for 4 min before going back to the initial ratio and maintained as such for 4 min before next injection. MassHunter Workstation v10.0 and ChemStation software were used for data collection and analysis.

Elution for targeted GA1 bioactive secondary metabolites analysis by UPLC MS were performed at 40 °C with a constant flow rate of 0.6 mL/min using a gradient of acetonitrile (solvent B) and water (solvent A) both acidified with 0.1% formic acid as follows: 2 min at 15% B followed by a gradient from 15% to 95% during 5 min and maintained at 95% up to 9.5 min before going back to initial conditions at 10 min during 2 min before next injection. Compounds were detected in both electrospray positive and negative ion mode by setting SQD parameters: cone voltage: 60V; source temperature 130 °C; desolvation temperature 400 °C, and nitrogen flow: 1000 L/h with a mass range from m/z 300 to 2048. MassLynx software v4.1 software was used for data collection and analysis.

**MZmine analysis**

Mzmine 2 parameters used in this study were setup as follow: MS 1 noise level was set at 1x10⁴. Further, the ADAP chromatogram builder was used (min group size in scans = 5, Group intensity threshold = 2x10⁴ and minimum highest intensity = 3x10⁴, m/z tolerance = 20 ppm). The chromatogram deconvolution was made by Local minimum search (chromatographic threshold = 5%, Search min Rt range = 0.15 min, Min relative height = 10%, Min absolute height = 3x10⁴, min ration of peak top/edge = 1.5, peak duration range = 0.1-1 min, m/z center calculation is the median, m/z range for MS2 pairing = 0.02 Da, Rt range for MS2 scan pairing = 0.2 min) while isotope peak grouper was setup as follow: m/z tolerance = 20 ppm, retention time tolerance = 0.2 min, maximum charge = 2. Further, the join aligner was using following parameters: m/z tolerance = 20 ppm, weight for m/z = 75, retention time tolerance = 0.2 min, weight for Rt = 25. The data was adapted with feature list filter (minimum peaks in a row = 3, minimum peaks in an isotope pattern = 2, Retention time range from 1.5 to 20 min).

**Purification of pyoverdine, E-PCH** **and PCH**

This part is in complement of the “Purification of pyoverdine, E-PCH and PCH” section of the Material and methods. The volume injected was 100 µL. The UV-Vis absorbance was measured with a VWD Agilent technologies 1100 series (G1314A) detector (Agilent, Waldbronn, Germany). The lamp used was a Deuterium lamp G1314 Var Wavelength Det. (Agilent, Waldbronn, Germany). Two wavelengths were selected: 320 nm, used for the detection of E-PCH and PCH, and 380 nm, used to detect pyoverdine. The fractions containing the siderophores were collected directly at the detector output. Further, the purity of the samples was verified by two detectors, a diode array detector (DAD) 190 to 601 nm (steps: 1 nm) and a q-TOF (tandem mass spectrometry, quadrupole, and Time of flight detector combined) (Agilent, Waldbronn, Germany). Electrospray ionization was performed in positive mode (ESI+) (Dual AJS ESI) (Vcap = 3500 V, Nozzle Voltage = 1000 V), with a mass range from m/z 200 to 1500.

The concentration of siderophores was estimated using Beer-Lambert law formula, A = Ɛlc (A: absorbance; Ɛ: molar attenuation coefficient or absorptivity of the attenuating species; l: optical path length and c: concentration of molecule). l value for E-PCH and pyoverdine is 1 cm while Ɛ is 4000 L.mol^-1^.cm^-1^ or 16000 L.mol^-1^.cm^-1^, respectively and as established previously [2]. The absorbance was measured with VWR, V-1200 Spectrophotometer, at 320 nm (pH = 8) for E-PCH or PCH and 380nm (pH = 5 ) for pyoverdine [2]. Further, the absorbance value was used for calculating the final concentration.

**SUPPLEMENTARY FIGURES**


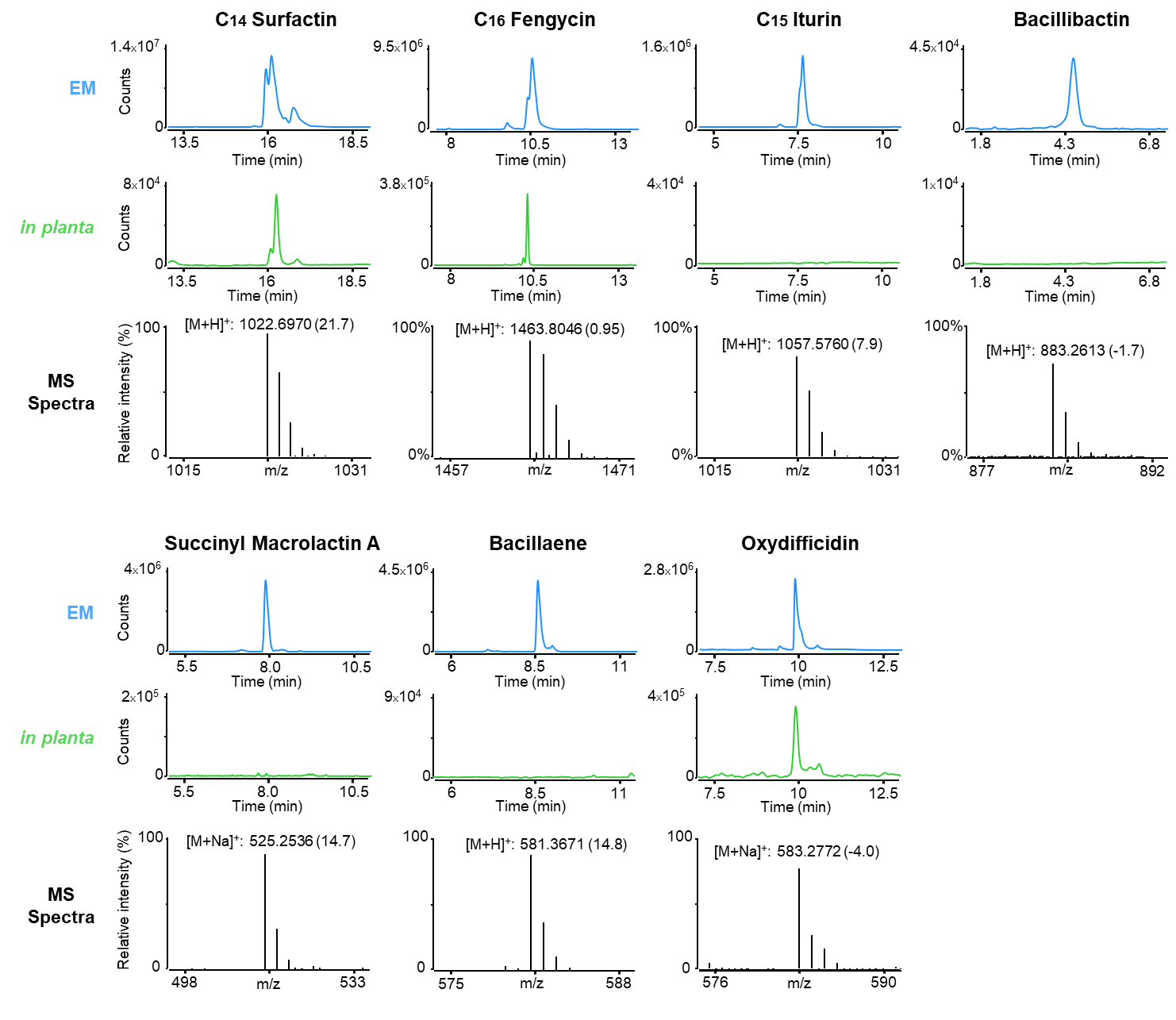


**Figure S1. The relative amount of** bioactive secondary metabolites **produced by *B. velezensis* GA1 in different conditions.** Extracted ion chromatograms (EICs) of the different compounds detected in 24 h liquid culture (EM: exudates mimicking medium) or recovered from agar surrounding colonized tomato plantlets at 7 dpi (*in planta*). Structural variant and MS spectra with ion species and mass error (in ppm) are mentioned for each metabolite.


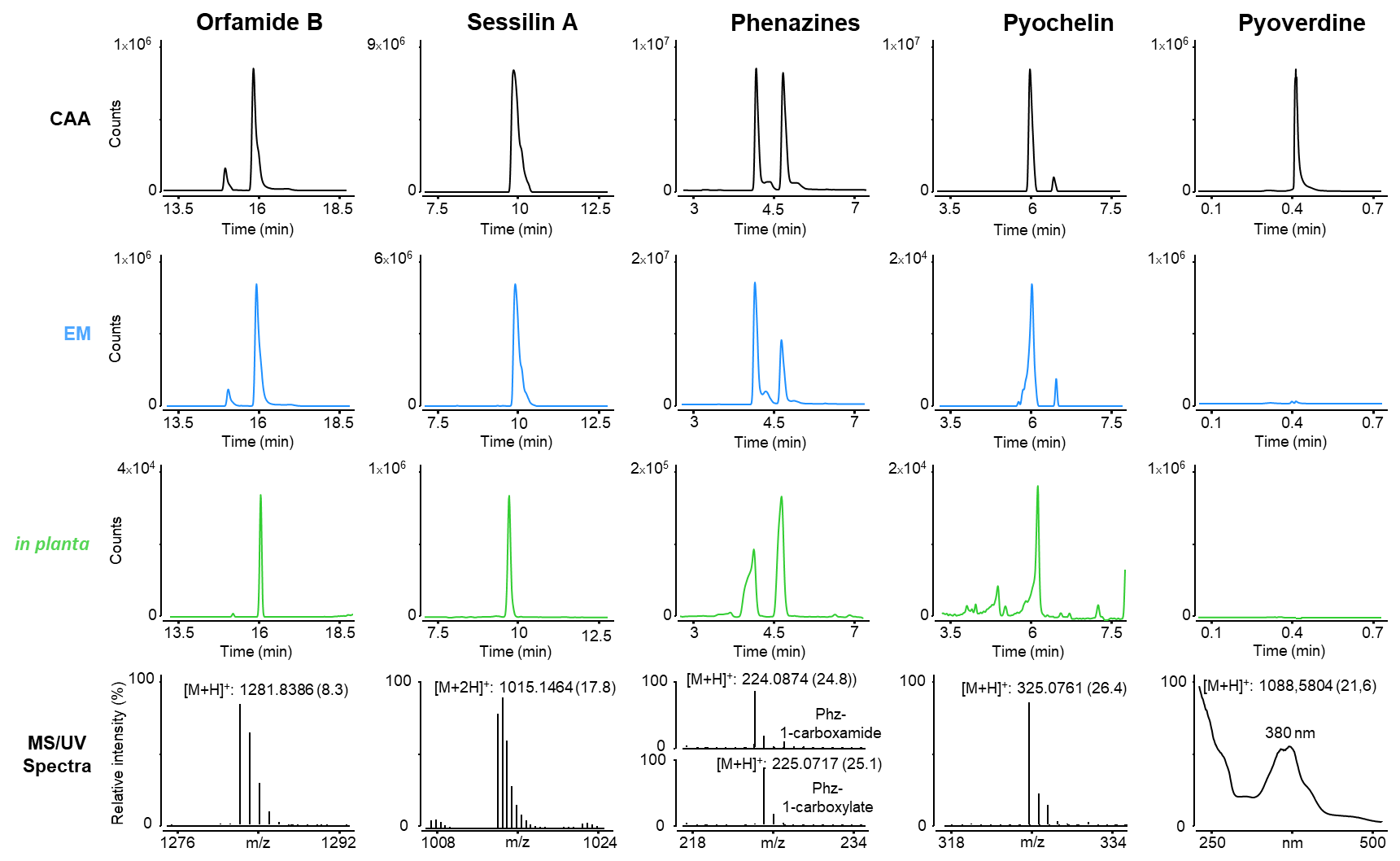


**Figure S2. The relative amount of bioactive secondary metabolites (BSMs) produced by *P. sessiligenes* CMR12a in different conditions.** Extracted ion chromatograms (EICs) of the different BSMs detected in 24 h liquid culture (CAA : casamino acids medium, EM: exudates mimicking medium) or recovered from agar surrounding colonized tomato plantlets at 7 dpi (*in planta*). Structural variants and MS/UV spectra with wavelength for pyoverdine and ion species with mass error (in ppm) are mentioned for each BSM.

**Figure S3. Effect of *P. sessiligenes* CMR12a metabolites on *B. velezensis* GA1 growth.** *B. velezensis* GA1 (GA1) growth upon supplementation of culture medium with 2% (v/v) *P. sessiligenes* CMR12a cell free supernatant (GA1 + CFS). Data show the mean and ± SD from three replicates.

**Figure S4. Effect of *P. sessiligenes* CMR12a supernatant on *B. velezensis* GA1 metabolite production.** GA1 production of iturins, fengycins, macrolactins, and bacilysin upon interaction with CMR12a cell free supernatant (2% (v/v)) during 15 h, compared to un-supplemented cultures (fold change = 1, red line). Production data are expressed as peak area fold change per OD_600_ and compared to control culture (un-supplemented GA1 culture). Error bars represent standard deviation (n = 3). Mean values were calculated from data obtained in three repeats (n = 3). Statistical significance was calculated using Mann–Whitney test where ‘’ns’’ represents a non-significant difference.

**Figure S5. Effect of *P. sessiligenes* CMR12a metabolites on bioactive secondary metabolites (BSMs) production by *B. velezensis* strains GA1, S499, FZB42, and QST713.** Data indicate fold increase in BSMs production upon addition of CMR12a cell free supernatant (2% v/v) compared to un-supplemented cultures (fold change = 1, red line). Data were calculated based on relative quantification of the compounds by UPLC-MS (peak area) per OD600 in both conditions. Mean values and ± SD were calculated from three cultures (repeats) in three independent experiments (n = 9). Statistical significance was calculated using Mann–Whitney test where “ns” means no significant difference; “*”, *p<*0. 1; ’’**’’, *p<*0.01; ‘’****’’, *p<*0.0001.


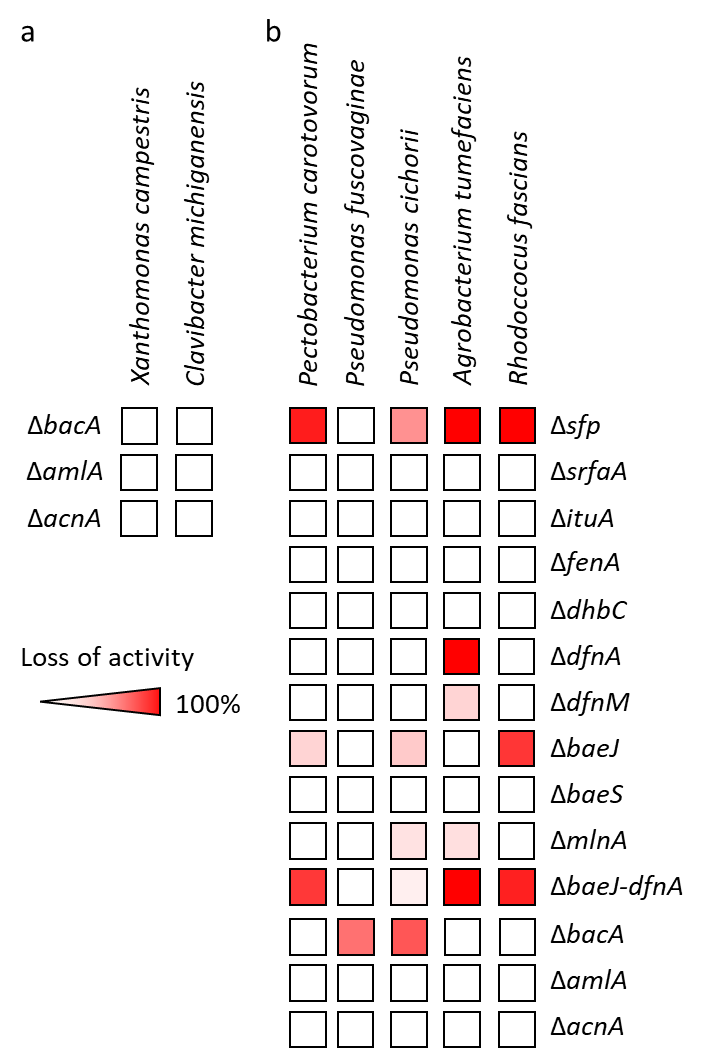


**Figure S6. The anti-bacterial activities of *B. velezensis* GA1 rely on the production of different** bioactive secondary metabolites **according to the target species. A,** The heatmap indicates retention of anti-*Xanthomonas* activity of GA1 mutants unable to produce bacilysin (*ΔbacA*), amylolysin (Δ*amlA*) and amylocyclicin (*ΔacnA*), or bacilysin and amylolysin against *Clavibacter*. **b,** The heatmap shows the loss of anti-bacterial activity of GA1 mutants compared to the wild-type strain when tested against five plant pathogenic species. The mutants are impaired in the production of non-ribosomal peptides and PKs (Δ*sfp*), surfactins (Δ*srfaA*), iturins (Δ*ituA*), fengycins (Δ*fenA*), difficidins (Δ*dfnA*), bacillaenes (Δ*baeJ*), difficidins and bacillaenes (Δ*baeJ-dfnA*), macrolactins (Δ*mlnA*), bacilysin (Δ*bacA*), amylolysin (Δ*amlA*), and amylocyclicin (Δ*acnA*). The intensity of activity loss is represented by the color scale where the darkest red indicates loss of 100% and white reflects no difference compared to the wild type. None of the mutants displayed gain in activity compared to the wild-type strain. Heatmap shows the mean of three biological replicates (n = 3).


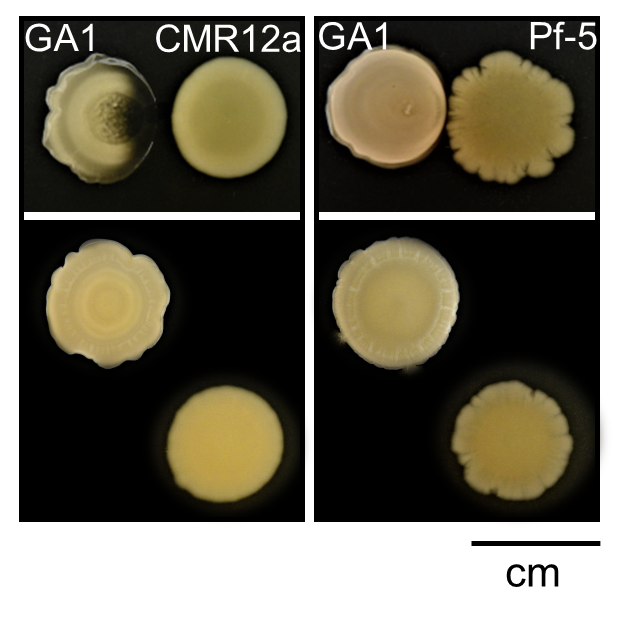


**Figure S7. *B. velezensis* GA1 does not inhibit *P. sessiligenes* CMR12a and *P. protegens* Pf-5.** The growth of CMR12a and Pf-5 during the confrontation with GA1 at a short distance (1 mm) (upper panel), compared with the growth of the strains alone (panel down). Data are from one representative of three independent replicates.

**Figure S8. Stimulation of amylocyclicin gene expression in *B. velezensis* strain S499 in response to *P. sessiligenes* CMR12a metabolites.** Relative expression of the *acnA* gene measured after 8 h of incubation in the presence of CMR12a cell free supernatant (CFS) (added at 2% v/v) compared to un-supplemented cultures (CTRL). Data show means and ± SD calculated from three cultures in two independent experiments (n = 6). The statistical difference, in *acnA* expression, between the two conditions was calculated using the T-test, “***”, *p<*0.001.

**
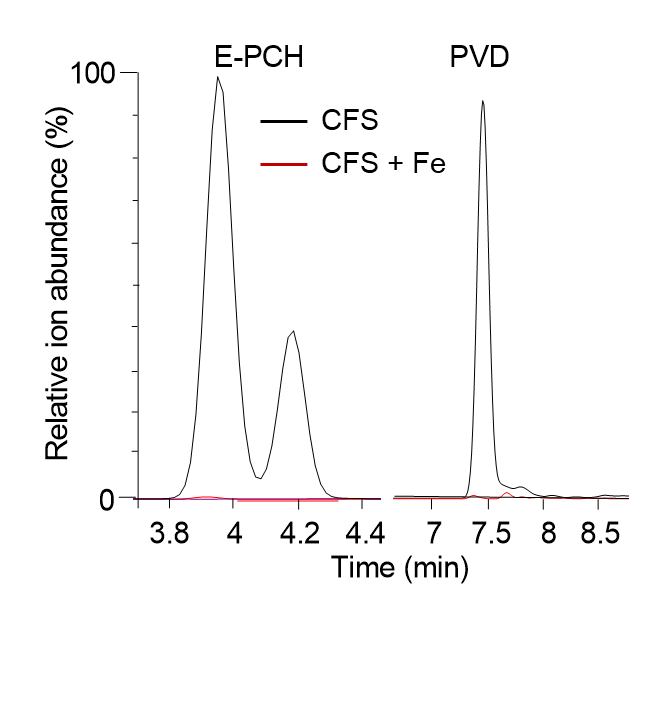
**

**Figure S9. The addition of iron into the culture medium represses siderophore production by *P. sessiligenes* CMR12a.** UPLC-qTOF MS extracted ions chromatograms illustrate the relative abundance of ions corresponding to pyoverdine (PVD) (m/z 1289.59 and enantio-pyochelin (E-PCH) (m/z 325.07) and as produced upon growth in casamino acids medium (black line) or casamino acids medium supplemented with 20 μg/L of FeCl₃x6H₂O (red line). The major peak at RT 3.95 min represents the mainPVD form described in Fig. S2, while the minor peak at RT 4.2 min corresponds to a structural variant with similar peptide moiety but most probably a different side-chain in the chromophore (not identified).

**Figure S10. Effect of pyoverdine (PVD) and enantio-pyochelin (E-PCH) on the growth of the bacillibactin-suppressed mutant of *B. velezensis* GA1 (CTRL).** Pure compounds were added to the GA1 culture at a concentration corresponding to the one resulting from the addition of cell free supernatant CAA at 2% (v/v). OD600 was measured at the mid-exponential phase. Data represent means and ± SD calculated from three replicate cultures in two independent experiments (n = 6), T-test with ’ns’ = no significant difference; “**”, statistically different at *p<*0.01.


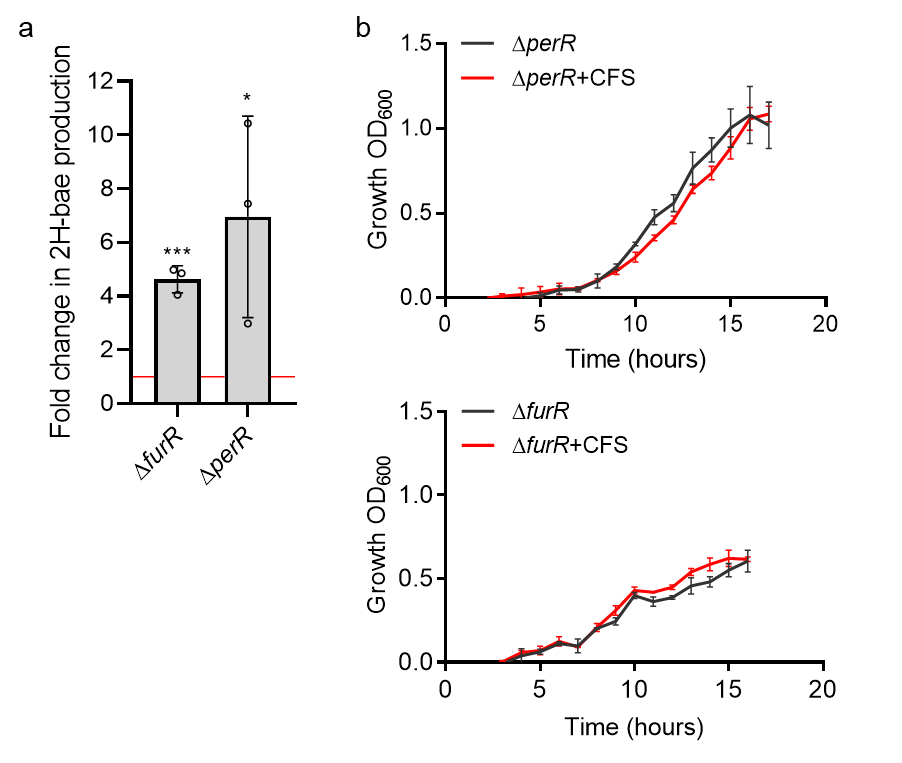


**Figure S11. E-PCH triggering activity mechanism is not related to iron- and oxidative-stress cause in *B. velezensis* GA1. a,** Dihydrobacillaene (2H-bae) production by GA1 mutants disrupted in ferric uptake (Δ*furR*) and oxidative-stress regulator (Δ*perR*) upon 2% (v/v) CMR12a cell free supernatant (CFS) supplementation in comparation with the corresponding, un-supplemented control (fold change = 1, labeled as a red line). Mean values and ± SD were calculated from three cultures (repeats) (n = 3). Statistical significance was calculated using Mann–Whitney test where “*”, *p<*0.1 and “***”, *p<*0.001. **b,** The growth of Δ*furR* and *ΔperR* upon supplementation of culture medium with 2% (v/v) of CMR12a CFS (Δ*furR*+CFS or Δ*perR*+CFS) in comparision with un-supplemented control (Δ*furR* or Δ*perR*). Means and ± SD are from three replicates.

**Figure S12: Stimulation of dihydrobacillaene production by enantio-pyochelin producers.** The dihydrobacillaene (2H-bae) production by GA1 upon culture supplementation with 2% (v/v) of CMR12a and Pf-5 cell free supernatant. Fold change equal to 1 is represented as a red line and corresponds to the production of 2H-bae in GA1 un-supplemented culture. The graph shows the mean and ± SD of three biological replicates with three technical repetitions (n = 9). Statistical significance was calculated using Mann–Whitney test where ‘’ns’’, not a significant difference and “****”, *p<*0.0001.


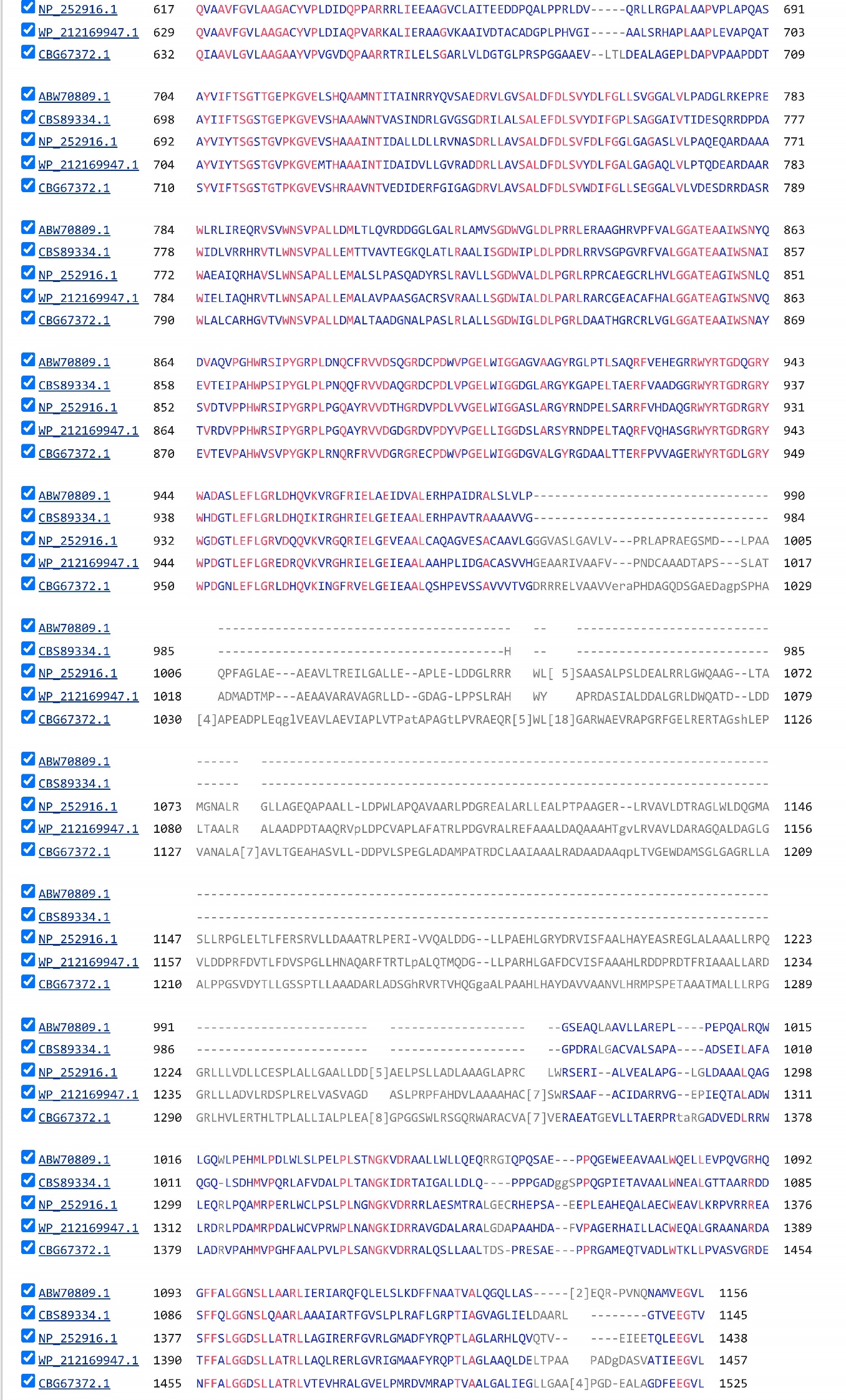

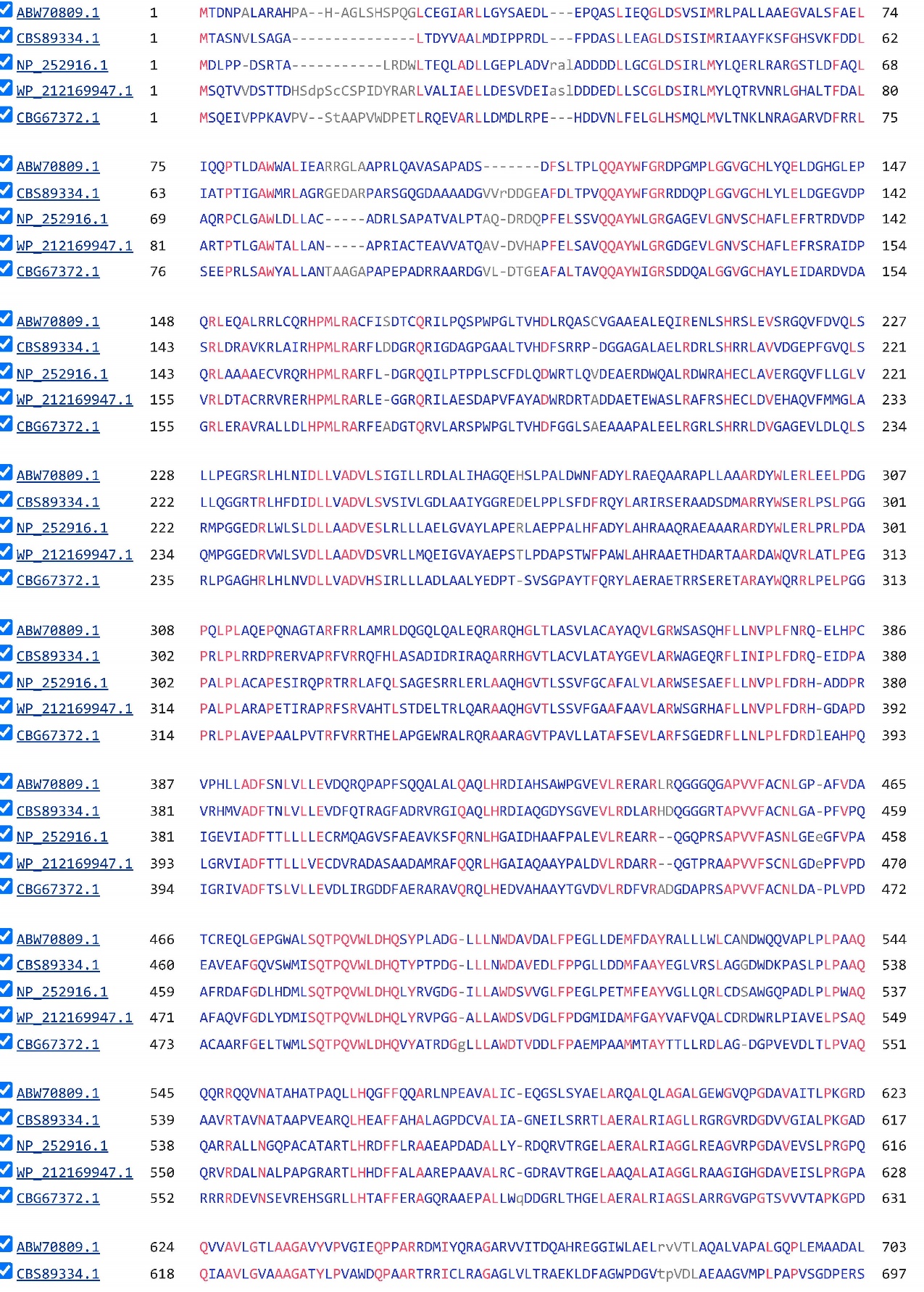

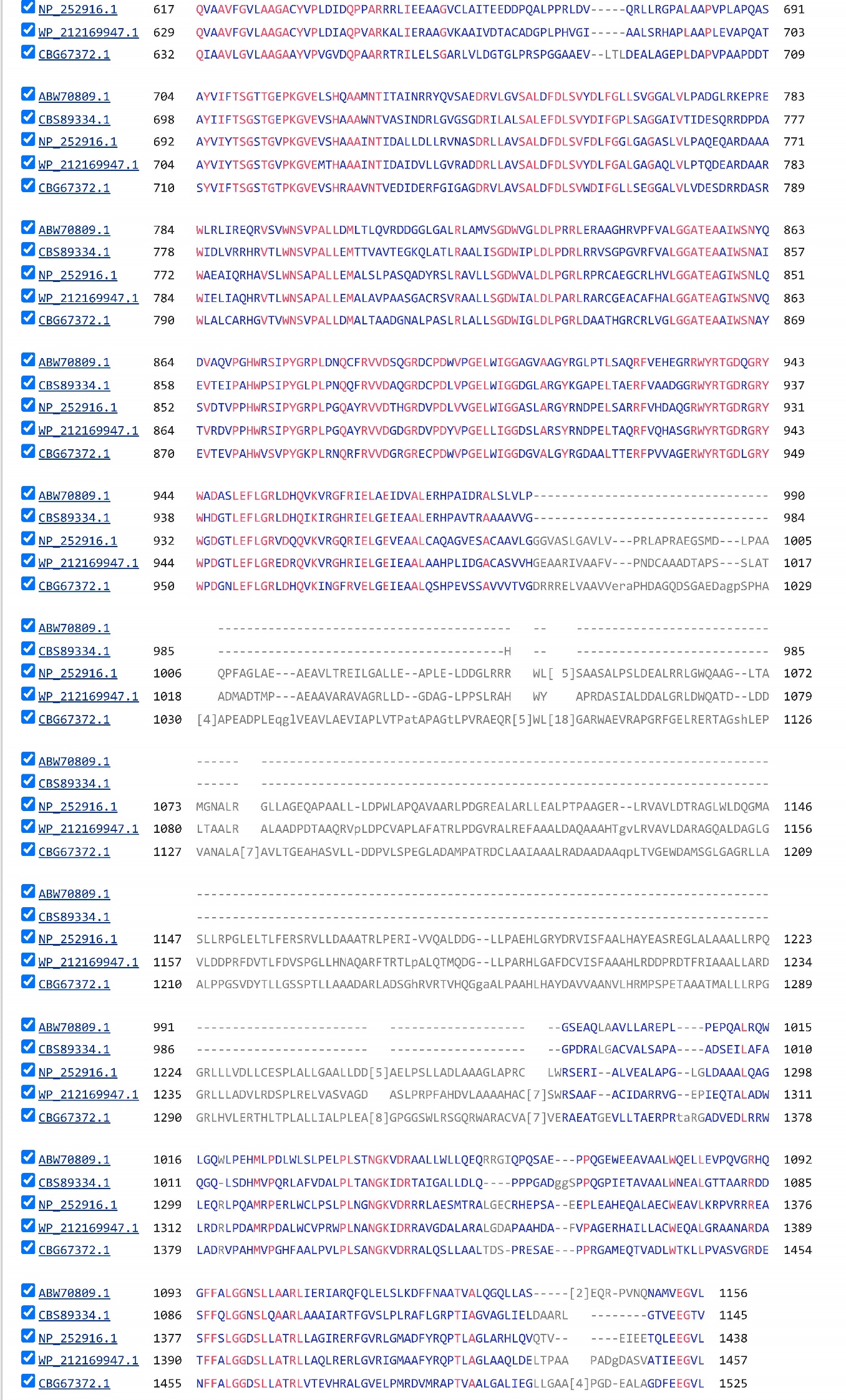


**Figure S13: Alignment of the PchE protein from several bacteria.** Sequences were aligned with the Constraint-based multiple alignment tool (COBALT) in NCBI. Sequence IDs are given. Multiple sequence alignment columns with no gaps are colored in blue or red. Amino acids in red are identical, amino acids in blue are less conserved. ABW70809.1: *Pseudomonas protegens* CHA0; CBS89334.1: *Azospirillum lipoferum* 4B; NP_252916.1; *Pseudomonas aeruginosa* PAO1; WP_212169947.1: *Burkholderia cenocepacia*; CBG67372.1: *Streptomyces scabiei* 87.22

**SUPPLEMENTARY TABLES**

**Table S1.** Strains and plasmids used in this study.

|  | **Relevant genotype and description** | **References or sources** |
| --- | --- | --- |
| ***B. velezensis*** |  |  |
| GA1 | Wild type | [3] |
| GA1 ∆*sfp* ::cat | GA1 deleted of *sfp* gene; unable to produce lipopeptides, polyketides and bacillibactin | This study |
| GA1 ∆*srfaA* ::cat | GA1 deleted of *srfaA* gene; unable to produce surfactins | [4] |
| GA1 ∆*ituA* ::cat | GA1 deleted of *ituA* gene; unable to produce iturins | This study |
| GA1 ∆*fenA* ::cat | GA1 deleted of *fenA* gene; unable to produce fengycins | This study |
| GA1 ∆*dhbC* ::cat | GA1 deleted of *dhbC* gene; unable to produce bacillibactin | This study |
| GA1 ∆*baeJ* ::cat | GA1 deleted of *baeJ* gene; unable to produce bacillaene and dihydrobacillaene | This study |
| GA1 ∆*baeS* ::cat | GA1 deleted of *baeS* gene; unable to produce bacillaene | This study |
| GA1 ∆*dfnA* ::cat | GA1 deleted of *dfnA* gene; unable to produce difficidin and oxydifficidin | This study |
| GA1 ∆*dfnM* ::cat | GA1 deleted of *dfnM* gene; unable to produce oxydifficidin | This study |
| GA1 ∆*baeJ* ::cat ∆*dfnA* ::phleo | GA1 deleted of *baeJ* and *dfnM* genes; unable to produce bacillaenes and difficidins | This study |
| GA1 ∆*mlnA* ::cat | GA1 deleted of *mlnA* gene; unable to produce macrolactins | This study |
| GA1 ∆*acnA* ::cat | GA1 deleted of *acnA* gene; unable to produce amylocyclicin | This study |
| GA1 ∆*acnA*::cat ∆*sfp*::phleo | GA1 deleted of *sfp* and *acnA* genes; unable to produce lipopeptides, polyketides, bacillibactin and amylocyclicin | This study |
| GA1 ∆*amlA* ::cat | GA1 deleted of *amlA* gene; unable to produce amylolysin | This study |
| GA1 ∆*bacA* ::cat | GA1 deleted of *bacA* gene; unable to produce bacilysin | This study |
| GA1 ∆*fur* ::cat | GA1 deleted of *fur* gene; devoted of FurR function | This study |
| GA1 ∆*per* ::cat | GA1 deleted of *per* gene; devoted of PerR function | This study |
| S499 | Wild type | [5] |
| FZB42 | Wild type | [6] |
| QST713 | Wild type | [7] |
| ***P. sessiligenes*** |  |  |
| CMR12a | Wild type | [8,9] |
| Δ*sesA* | CMR12a disrupted of *sesA* gene; Gm^R^; unable to produce sessilins | [10] |
| Δ*ofaBC* | CMR12a deleted of *ofaB* and *ofaC* genes; Gm^R^; unable to produce orfamides | [11] |
| Δ*phz* | CMR12a deleted of phenazine biosynthesis operon; unable to produce phenazines | [10] |
| Δ*sesA-ofaBC* | CMR12a disrupted of *sesA* gene and deleted *ofaB* and *ofaC* genes; Gm^R^; unable to produce sessilins and orfamides | [11] |
| Δ*sesA-phz* | CMR12a disrupted of *sesA* gene and deleted phenazines biosynthesis operons; Gm^R^; unable to produce sessilins and phenazines | [10] |
| Δ*ofaAC-phz* | CMR12a deleted of *ofaB* and *ofaC* genes and phenazines biosynthesis operons; unable to produce orfamides and phenazines | [11] |
| Δ*sesA-ofaBC-phz* | CMR12a disrupted of *sesA* gene and deleted *ofaB* and *ofaC* genes and phenazine biosynthesis operons; Gm^R^; unable to produce sessilins, orfamides and phenazines | [11] |
| Δ*pchA* | CMR12a deleted of *pchA* gene; unable to produce enantio-pyochelin | [4] |
| Δ*pvdI* | CMR12a deleted of *pvdI* gene; unable to produce pyoverdine | [4] |
| Δ*pvdI-pchA* | CMR12a deleted of *pvdI* and *pchA* genes; unable to produce pyoverdine and enantio-pyochelin | [4] |
| ***P. protegens*** |  |  |
| Pf-5 | Wild type; enantio-pyochelin producer | [12] |
| ***P. aeruginosa*** |  |  |
| PAO1 | Wild type; pyochelin producer | [13] |
| ***E. coli*** |  |  |
| *DH5αpir* | supE44, ΔlacU169 (ΦlacZΔM15), recA1, endA1, hsdR17,  thi-1, gyrA96, relA1, λpir | [14] |
| *DH5α p497* | Helper strain harboring P497 plasmid | C. Keel laboratory |
| **Plasmids** |  |  |
| pEMG | pSEVA212S; oriR6K, *lacZα* with two flanking I-SceI sites; Km^r^, Ap^r^ | [15] |
| pEMG-*pchA* | Suicide plasmid used for the deletion of *pchA* | This study |
| pEMG-*pvdI* | Suicide plasmid used for the deletion of *pvdI* | This study |
| pSW-2 | oriRK2, *xylS*, *P_m_::I-sceI*; Gm^R^ | [15] |
| **Phytopathogenic strains** |  |  |
| *Xanthomonas campestris* pv. *campestris* LMG 582 |  | De Mot laboratory |
| *Clavibacter michiganensis* subsp. *michiganensis* |  | De Mot laboratory |
| *Pectobacterium carotovorum* LMG 6663 |  | De Mot laboratory |
| *Pseudomonas fuscovaginae* LMG 2158 |  | De Mot laboratory |
| *Pseudomonas cichorii* LMG 2162 |  | De Mot laboratory |
| *Agrobacterium tumefaciens* C58 |  | De Mot laboratory |
| *Rhodococcus fascians* D-188 |  | De Mot laboratory |

**Table S2.** Primers used in this study.

|  | **Primer Name** | **Primer sequence (5'->3')** | **Targeted genes** |
| --- | --- | --- | --- |
| **Deletion mutant** |  |  |  |
| ***B. velezensis* GA1** |  |  |  |
|  | UpsrfaAF | TCAGCAAAACTGCGTGGTAG | *srfaA* |
|  | UpsrfaAR | CCAATTTTCGAATTCTTTTACCGCGATAAAAAGTTATTTCCATATGTGTGC |  |
|  | DwsrfAF | CAGCTCCAGATCCTCTACGCCGGACACGCTTTATATCGTGCCGAA |  |
|  | DwsrfAR | AAGAAATGATCATAAATACC |  |
|  | UpFenAF | AGCAAAAACCGGGTCACTAA | *fenA* |
|  | UpFenAR | CCAATTTTCGAATTCTTTTACCGCGTTCGTCTGACATGACAAGCA |  |
|  | DwFenAF | CAGCTCCAGATCCTCTACGCCGGACAAAGGACTTTAATTTCATAAAAAGGTG |  |
|  | DwFenAR | CCTTTTTGAGAAGAGAAGAAAAAG |  |
|  | UpItuAF | ATGCAGGAAATAGGGGTGAA | *ituA* |
|  | UpItuAR | CCAATTTTCGAATTCTTTTACCGCGGGTATACATAGGTCCCCTCCTG |  |
|  | DwItuAF | CAGCTCCAGATCCTCTACGCCGGACCAATTGAACTTTTAGGGAAAAGCA |  |
|  | DwItuAR | GCGACTAACGTATCGGGTTG |  |
|  | UpDfnAF | GACTTTTGAATAATCTACAGTGTCTCC | *dfnA* |
|  | UpDfnAR | TTTTCGAATTCTTTTACCGCGAAACGCGTTTGCGATTCAG |  |
|  | UpDfnAphleoR | CAGGAAACAGCTATGACAAACGCGTTTGCGATTCAG |  |
|  | DwDfnAphleoF | GTAAAACGACGGCCAGTACAGGCTGAGTATGACCAGACA |  |
|  | DwDfAF | CAGCTCCAGATCCTCTACGCCGGACACAGGCTGAGTATGACCAGACA |  |
|  | DwDfnAR | TCCGGAATATGATCTTGTGAAG |  |
|  | UpDfnMF | GGGCAGTGGAGCTGTACC | *dfnM* |
|  | UpDfnMR | CCAATTTTCGAATTCTTTTACCGCGGGTCATTTTCATTCCTCCAAGA |  |
|  | DwDfnMF | CAGCTCCAGATCCTCTACGCCGGACCTTGTTGAGTTTTGAACGAAAAA |  |
|  | DwDfnMR | AGCCGTTATCAATCGTGCTG |  |
|  | UpBaeJF | GTATGCGTCCCAGACTCAGC | *baeJ* |
|  | UpBaeJR | CCAATTTTCGAATTCTTTTACCGCGTTTCATAGAGCTGCCTCCAT |  |
|  | DwBaeJF | CAGCTCCAGATCCTCTACGCCGGACGGGATACCTATGAAGTGGAGGTT |  |
|  | DwBaeJR | TCATAGTAGCCGACTTGAGAATCA |  |
|  | UpBaeSF | GTACAGCAAGGTGCCATGAG | *baeS* |
|  | UpBaeSR | CCAATTTTCGAATTCTTTTACCGCGTTTTTGAAAAGACATAACCAACAG |  |
|  | DwBaeSF | CAGCTCCAGATCCTCTACGCCGGACTTTTAATATCGCCCCCTGTTT |  |
|  | DwBaeSR | GAGGCGTTGAAGCATACCAG |  |
|  | UpsfpF | TCGTCACCCATGAAATCAAA | *sfp* |
|  | UpsfpR | CCAATTTTCGAATTCTTTTACCGCGCATGTCCAGATCCTCCGTCT |  |
|  | DwsfpF | CAGCTCCAGATCCTCTACGCCGGACGACGGGATTGAGATGAAAA |  |
|  | DwsfpR | CATTGAGACGTACCCGCTTT |  |
|  | UpdhbCF | GCGTTTCTGCCTGAATCC | *dhbC* |
|  | UpdhbCR | CCAATTTTCGAATTCTTTTACCGCGCATGTTTGTCCCTCCTTTTCGT |  |
|  | DwdhbCF | CAGCTCCAGATCCTCTACGCCGGACGGCTTTACCAAGATGA |  |
|  | DwdhbCR | GCAGCACTTGAAGGCTTGAT |  |
|  | UpmlnAF | CGGAAAAACCGTTTCAAAAA | *mlnA* |
|  | UpmlnAR | CAGGAAACAGCTATGACTTTTAAAATTGTCATTTACTCTAAGCA |  |
|  | DwmlnAF | GTAAAACGACGGCCAGTCTAAGGCGCAGATTGGATA |  |
|  | DwmlnAR | TGTACCTGTGCCATGTGCTT |  |
|  | UpbacAF  UpbacAR  DwbacAF  DwbacAR | GATGGGTCTGATCGTGTCAA  CCAATTTTCGAATTCTTTTACCGCGCATGAGCACCAACCAATCTG  CAGCTCCAGATCCTCTACGCCGGACAACTGAACAAGATTTGCAGG  GAATCGGGGCGACAATTT | *bacA* |
|  | UpacnAF | TCCTTGTCACTGGGTGATGA | *acnA* |
|  | UpacnAR | TTTCGAATTCTTTTACCGCGGTTCATCATAACATCTCCCTACTCTG |  |
|  | DwacnAF | CCAGATCCTCTACGCCGGACGCAGCTGCTTGGTAAAATCG |  |
|  | DwacnAR | CGCAAAATCAGCGTTTGTC |  |
|  | UpamlAF | GGGCTGACAGGGATAAAAGA | *amlA* |
|  | UpamlAR | TTTCGAATTCTTTTACCGCGCTCATTCATTAATATTCCTCCCTTTG |  |
|  | DwamlAF | CCAGATCCTCTACGCCGGACTGGTGTTAAAACAACCCGAAA |  |
|  | DwamlAR | TCATGATCTCTAATTTTCTCATTCAAA |  |
|  | UpfurF | TCTCAGAAGAAACGGGATGC | *fur* |
|  | UpfurR | CAGGAAACAGCTATGACCATGTCTTTCCCTCCTACGC |  |
|  | DwfurF | GTAAAACGACGGCCAGTTAAAAGCCTATGAACCTTTTCTGC |  |
|  | DwfurR | ATCCCTGACGGCTGATCT |  |
|  | UpperRF | GTACAGCACGCCGTTTTCC | *per* |
|  | UpperRR | CAGGAAACAGCTATGACCATCCGTCATGCACCTCTC |  |
|  | DwperRF | GTAAAACGACGGCCAGTAAAGAAAACCACTAAACGAAGCTG |  |
|  | DwperRR | CGCTTTTCCTTCTACAAATCTTC |  |
| ***P. sessiligenes* CMR12a** |  |  |  |
|  | UppvdIF | GGCATTCTTGACCGGTCGTC | *pvdI* |
|  | UppvdIR | GTGTTGTCCATTACACAGCCTCCATTGCATTCATCGGGAGTCATCC |  |
|  | DwpvdIF | ATGGAGGCTGTGTAATGGACAACA |  |
|  | DwpvdIR | TGTAGCGGTGTAGCAGAG |  |
|  | pvdICheckF | CCTGCTGCTGGAAGGATTGA |  |
|  | pvdICheckR | GGATCGAGCTGCCAAAGGAA |  |
|  | UppchAF | GACCAACTGCCGGCGGAT | *pchA* |
|  | UppchAR | CCTTCAGCGATCGGCCGGTGCATCACATCTTGCGCTCCTTGCTCC |  |
|  | DwpchAF | TGATGCACCGGCCGATC |  |
|  | DwpchAR | GTGGTGAAGCTTTCCATGCC |  |
|  | pchACheckF | TCATCCACTGGAACATCGCC |  |
|  | pchACheckR | GCGGACTGATTTCCTCGGTA |  |
| **Antibiotic marker** |  |  |  |
|  | CatF | CGCGGTAAAAGAATTCGAAAA | Chloramphenicol marker |
|  | CatR | GTCCGGCGTAGAGGATCTG |  |
|  | PhleoF | GTCATAGCTGTTTCCTGCCAAAAGGGGGTTTCATTTT | Phleomycin marker |
|  | PhleoR | ACTGGCCGTCGTTTTACTCCAATAAATGCGACACCAA |  |
|  | nptIIF | GAGGATCGTTTCGCATGATT | Kanamycin marker for *Pseudomonas* |
|  | nptIIR | CGCTCAGAAGAACTCGTCAA |  |
|  | psw-F | GGACGCTTCGCTGAAAACTA | pSW-II insertion |
|  | psw-R | AACGTCGTGACTGGGAAAAC |  |
| **RT-qPCR** |  |  |  |
| ***B. velezensis* GA1** |  |  |  |
|  | AcnA_F_qPCR | CCAAGCAGCTGCGTATTTTT | *acnA* |
|  | AcnA_R_qPCR | CTTCGACTCTGGGCATCTCT |  |
|  | QgyrA_F | GAGACGCACTGAAATCGTGA | *gyrA* |
|  | QgyrA_R | GCCGGGAGACGTTTAACATA |  |
|  | BaeJQ_F_qPCR | CCGATGACGATTCCTGAAGT | *baeJ* |
|  | BaeJQ_R_qPCR | GCCCTTTCACAATCGAAAGA |  |
|  | DfnA_F_qPCR | GGCGTTTTTGCCTCTTCGTT | *dfnA* |
|  | DfnA_R_qPCR | ATCAGACGGCGTATCGTGTC |  |
|  | SrfaA_F_qPCR | ATTGTTTACGGTGGCTCTGG | *srfaA* |
|  | SrfaA_R_qPCR | CGCTGCGATAGTCAAAATCA |  |
|  |  |  |  |

**Table S3: Conservation of genes encoding substrate-binding proteins involved in iron transport in *B. subtilis* and *B. velezensis.*** The percentage of genes nucleotide identity was obtained by blast comparison performed on the MAGE platform (https://mage.genoscope.cns.fr).

|  | ***B. velezensis* GA1** | | ***B. velezensis* QST713** | | ***B. velezensis* S499** | | ***B. velezensis* FZB42** | |
| --- | --- | --- | --- | --- | --- | --- | --- | --- |
| **Transporters in *B. subtilis* 168** | **Gene name** | **% of identity** | **Gene name** | **% of identity** | **Gene name** | **% of identity** | **Gene name** | **% of identity** |
| *feuA* | *GL331_06035* | 83 | *BVQ_00990* | 83 | AS588_13145 | 83 | RBAM_002120 | 83 |
| *fhuD* | *GL331_01180* | 81 | *BVQ_17365* | 82 | AS588_15255 | 82 | RBAM_030440 | 82 |
| *yxeB (frxB)* | *GL331_04235* | 72 | *BVQ_20555* | 73 | AS588_18310 | 72 | RBAM_036560 | 72 |
| *fpiA* (*pbtQ/yclQ)* | *GL331_06955* | 74 | *BVQ_01985* | 73 | AS588_12230 | 74 | RBAM_004080 | 73 |
| *yfmC (fecC)* | *GL331_10185* | 36 | *BVQ_05570* | 36 | AS588_10320 | 36 | RBAM_010510 | 37 |
| *yfiY(sxzY)* | *GL331_04235 (yxeB)* | 33 | *BVQ_20555 (yxeB)* | 32 | AS588_18310 (*yxeB*) | 33 | RBAM_036560 (*yxeB*) | 32 |

**Table S4. The putative Fur box in the genome of *B. velezensis* GA1.** The putative position of Fur box (palindrome sequence: TGANAATNATTNTCA) in GA1 genome was obtained by nucleotide blast performed on the NCBI platform (https://blast.ncbi.nlm.nih.gov).

| **Found sequence** | **Sequence score (14)** | **Upstream gene (bp)** | **Downstream gene (bp)** |
| --- | --- | --- | --- |
| tgataatgattatca | 14 | Bacillibactin trilactone hydrolase (20) | 2,3-dihydro-2,3-dihydroxybenzoate dehydrogenase (99) |
| tgataatgattttca | 13 | 2,3-dihydro-2,3-dihydroxybenzoate dehydrogenase (43) |  |
| tgataatgattatca | 14 | iron ferrichrome abc transporter (35) | iron hydroxamate abc transporter (170) |
| tgaaaatgattctca | 12 | DNA methyladenine glycosylase (148) | Iron ABC transporter (48) |
| tgataatcattatca | 14 | hypothetical prot (94) | iron hydroxamate ABC transporter (19) |
| tgagaatcattatca | 13 | Fur regulated basic protein (RNA chaperone for fsrA (regulatory ncRNA) (25) | cation facilitator family transporter (173) |
| tgaaaatgattctca | 12 | heme degrading oxygenase (45) |  |
| tgataataattttca | 13 |  | DoxX family protein (linked to oxidative stress) (11) |
| tgataatcattttca | 13 |  | nitroreductase (23) |
| tgataattattatca | 14 |  | heavy metal translocating ATPase (162) |
| tgataatcattatca | 14 |  | DUF1797 family prot (domain unknown function) (360) |
| tgaaaatcattatca | 14 |  | flavodoxin (53) |
| tgaaaatcattatca | 14 |  | flavodoxin (15) |
| tgagaatcattttca | 12 | fbpC fur regulated basic protein (18) |  |
| tgagaatgattttca | 12 | fbpC fur regulated basic protein (53) |  |

**SUPPLEMENTARY REFERENCES**

1. Livak KJ, Schmittgen TD. Analysis of relative gene expression data using real-time quantitative PCR and the 2-ΔΔCT method. Methods. 2001;25:402–408.

2. Hoegy F, Mislin GLA, Schalk IJ. Pyoverdine and pyochelin measurements. Methods Mol Biol. 2014;1149:293–301.

3. Touré Y, Ongena M, Jacques P, Guiro A, Thonart P. Role of lipopeptides produced by *Bacillus subtilis* GA1 in the reduction of grey mould disease caused by *Botrytis cinerea* on apple. J Appl Microbiol. 2004;96:1151–1160.

4. Andrić S, Meyer T, Rigolet A, Prigent-Combaret C, Höfte M, Balleux G, et al. Lipopeptide interplay mediates molecular interactions between soil bacilli and pseudomonads. Microbiol Spectr. 2021;9:e0203821.

5. Molinatto G, Puopolo G, Sonego P, Moretto M, Engelen K, Viti C, et al. Complete genome sequence of *Bacillus amyloliquefaciens* subsp. *plantarum* S499, a rhizobacterium that triggers plant defences and inhibits fungal phytopathogens. J Biotechnol. 2016;238:56–59.

6. Chen XH, Koumoutsi A, Scholz R, Eisenreich A, Schneider K, Heinemeyer I, et al. Comparative analysis of the complete genome sequence of the plant growth-promoting bacterium *Bacillus amyloliquefaciens* FZB42. Nat Biotechnol. 2007;25:1007–1014.

7. Pandin C, Le Coq D, Deschamps J, Védie R, Rousseau T, Aymerich S, et al. Complete genome sequence of *Bacillus velezensis* QST713: A biocontrol agent that protects *Agaricus bisporus* crops against the green mould disease. J Biotechnol. 2018;278:10–19.

8. Perneel M, Heyrman J, Adiobo A, De Maeyer K, Raaijmakers JM, De Vos P, et al. Characterization of CMR5c and CMR12a, novel fluorescent *Pseudomonas* strains from the cocoyam rhizosphere with biocontrol activity. J Appl Microbiol. 2007;103:1007–1020.

9. Girard L, Lood C, Höfte M, Vandamme P, Rokni-Zadeh H, van Noort V, et al. The ever-expanding *Pseudomonas* genus: Description of 43 new species and partition of the *Pseudomonas putida* group. Microorganisms. 2021;9:1766.

10. D’aes J, Hua GKH, De Maeyer K, Pannecoucque J, Forrez I, Ongena M, et al. Biological control of *Rhizoctonia* root rot on bean by phenazine- and cyclic lipopeptide-producing *Pseudomonas* CMR12a. Phytopathology. 2011;101:996–1004.

11. D’aes J, Kieu NP, Léclère V, Tokarski C, Olorunleke FE, De Maeyer K, et al. To settle or to move? The interplay between two classes of cyclic lipopeptides in the biocontrol strain *Pseudomonas* CMR12a. Environ Microbiol. 2014;16:2282–2300.

12. Paulsen IT, Press CM, Ravel J, Kobayashi DY, Myers GSA, Mavrodi D V., et al. Complete genome sequence of the plant commensal *Pseudomonas fluorescens* Pf-5. Nat Biotechnol. 2005;23:873–878.

13. Stover CK, Pham XQ, Erwin AL, Mizoguchi SD, Warrener P, Hickey MJ, et al. Complete genome sequence of *Pseudomonas aeruginosa* PA01, an opportunistic pathogen. Nature. 2000;406:959–964.

14. Hanahan D. Studies on transformation of *Escherichia coli* with plasmids. J Mol Biol. 1983;166:557–580.

15. Martínez-García E, de Lorenzo V. Engineering multiple genomic deletions in Gram-negative bacteria: Analysis of the multi-resistant antibiotic profile of *Pseudomonas putida* KT2440. Environ Microbiol. 2011;13:2702–2716.
